# The plastid metalloprotease FtsH6 and small heat shock protein HSP21 jointly regulate thermomemory in *Arabidopsis*

**DOI:** 10.1038/ncomms12439

**Published:** 2016-08-26

**Authors:** Mastoureh Sedaghatmehr, Bernd Mueller-Roeber, Salma Balazadeh

**Affiliations:** 1 University of Potsdam, Institute of Biochemistry and Biology, Karl-Liebknecht-Straße 24-25, Haus 20, 14476 Potsdam-Golm, Germany; 2 Max Planck Institute of Molecular Plant Physiology, Cooperative Research Group, Am Mühlenberg 1, 14476 Potsdam-Golm, Germany

## Abstract

Acquired tolerance to heat stress is an increased resistance to elevated temperature following a prior exposure to heat. The maintenance of acquired thermotolerance in the absence of intervening stress is called ‘thermomemory' but the mechanistic basis for this memory is not well defined. Here we show that *Arabidopsis* HSP21, a plastidial small heat shock protein that rapidly accumulates after heat stress and remains abundant during the thermomemory phase, is a crucial component of thermomemory. Sustained memory requires that HSP21 levels remain high. Through pharmacological interrogation and transcriptome profiling, we show that the plastid-localized metalloprotease FtsH6 regulates HSP21 abundance. Lack of a functional FtsH6 protein promotes HSP21 accumulation during the later stages of thermomemory and increases thermomemory capacity. Our results thus reveal the presence of a plastidial FtsH6–HSP21 control module for thermomemory in plants.

## Abstract (web summary)

Exposure of plants to heat can promote increased tolerance to subsequent heat stress. Here, the authors show that prolonged expression of *Arabidopsis* small heat shock protein HSP21 promotes this thermomemory effect and that HSP21 levels are regulated by the plastid metalloprotease FtsH6 during the memory period.

## Graphical abstract

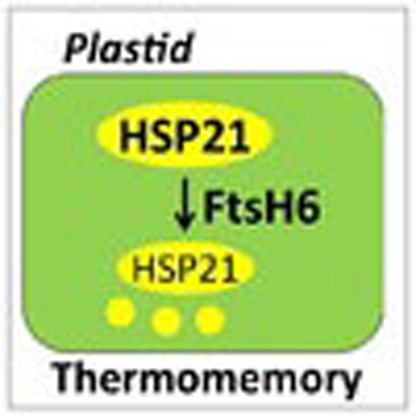

In their natural environment, plants are exposed to recurrent, sometimes irregular, environmental changes. Appropriate responses to environmental cues are required as plants cannot change their location on imposition of stress. Plants have an inherent ability to survive certain levels of stress (called basal tolerance), a characteristic that varies between species and genotypes. In addition, plants have the ability to acquire tolerance to otherwise lethal stresses and experimental evidence indicates the existence of a molecular ‘memory' that enables them to withstand stress better if previously confronted with the same or a similar type of stress^1^,^2^. Pre-exposure to stress, called priming, induces the configuration of a new cellular state (through molecular and biochemical recasting) that is different from the (pre-stress) naive situation and allows the plant to respond in a superior manner to subsequent stimuli (called triggering). The intervening time, during which plants experience a non-stress situation, is called the ‘memory' phase and can range from several hours to days, or even generations. However, the molecular machinery that underlies stress memory in plants is so far largely unknown.

An important environmental factor that often impairs plant growth, survival and productivity is heat. Heat stress affects the integrity of the proteome by causing misfolding and/or denaturation of proteins, thereby negatively affecting cell viability. Misfolded proteins are toxic to the cell and must be refolded, degraded or delivered to distinct quality control compartments that sequester potentially harmful misfolded proteins^3^.

Heat shock proteins (HSPs) are molecular chaperones that perform major roles in protecting the proteome against environmental stresses^4^. They are grouped into different families, based on their molecular masses: HSP70, HSP90, HSP100/ClpB (Hsp101) and small HSP (sHSP) families. SHSPs with a monomer molecular mass ranging from 12 to 42 kDa belong to an evolutionary conserved family harbouring a common α-crystallin domain located in the C-terminal part^5^. They occur in all eukaryotic organisms but are particularly abundant in plants, such as *Arabidopsis thaliana* and rice (*Oryza sativa*) which have 19 and 23 sHSPs, respectively, while there are 10 sHSPs in humans, 4 in *Drosophila melanogaster*, and 1 or 2 in bacteria^6^,^7^. The activity of sHSPs is independent of ATP and they are unable to refold non-native proteins, however, sHSPs provide immediate protection against unfavourable conditions by selectively binding to unfolded proteins and facilitating their subsequent refolding by other, ATP-dependent chaperones^6^,^7^,^8^,^9^,^10^. Hence, sHSPs play a critical role in the protein quality control system. Heat stress-induced expression of *HSP* genes is mainly controlled by heat shock transcription factors (HSFs) although other transcription factors also play a role^11^,^12^. In plants, sHSPs are targeted to different cellular compartments, including the cytosol, chloroplasts, mitochondria, and the endoplasmic reticulum^13^,^14^. Organelle-targeted sHSPs are unique to plants, the only known exception being mitochondrion-targeted sHSP22 in *Drosophila*^15^,^16^. Several sHSPs were shown to have important functions in the response of plants to heat stress and in acquired heat stress tolerance^6^,^10^,^17^.

The nuclear-encoded chloroplast protein HSP21 (monomer size 21 kDa, also known as HSP25.3-P (ref. 18)) is a plastid-localized sHSP in *Arabidopsis*^19^. It harbours a unique region towards its N terminus with several conserved methionine residues proposed to recognize hydrophobic stretches in partially unfolded proteins, and is mainly associated with thylakoid membranes^20^,^21^. Under conditions of oxidative stress the methionines are oxidized, and this process is reversed by a plastid-localized methionine sulfoxide reductase, which restores the chaperone activity of HSP21 (ref. 22). Under optimal growth conditions, *HSP21* expression is largely restricted to pollen grains but its expression rapidly and strongly increases in most organs on high-temperature stress^21^,^23^. Overexpression of *HSP21* in transgenic *Arabidopsis* plants enhances tolerance towards heat stress when imposed together with high light, a combination known to cause oxidative stress, while thermotolerance was not significantly altered in a low-light regime^19^; however, HSP21 is essential for chloroplast and early seedling development in the presence of heat stress^21^. In tomato (*Solanum lycerpersicum*), HSP21 was reported to protect photosystem II (PSII) on exposure of the plant to excessive temperature^24^.

Expression of *HSP21* is controlled by Heat shock factor A2 (HsfA2), a master transcriptional regulator of thermomemory in *Arabidopsis*. and the *hsfa2* knockout mutant is defective in thermomemory^25^. *HSP21* transcript is absent 24 h after heat priming in the *hsfa2* mutant while it is still highly abundant in wild-type plants. Absence of *HSP21* transcript is also evident in the *rof1* knockout mutant whose defective thermomemory phenotype resembles that of *hsfa2* (ref. 26). *ROF1* (*AtFKBP62*) encodes an FK506-binding protein-type peptidyl prolyl *cis*/*trans* isomerase. Similar to HsfA2, ROF1 is required for extending the duration of acquired thermotolerance (thermomemory), but not for its induction^26^.

Besides chaperones, proteases play a regulatory role in maintaining proteome homoeostasis in most subcellular compartments. Four major families of proteases; Clp, FtsH, DegP (in chloroplasts) and Lon protease (in chloroplasts and mitochondria)^27^, have been characterized in *Arabidopsis* and act as central elements of chloroplast and mitochondrial protein quality control systems^28^.

A relatively well-characterized family of proteases is the FtsH (filamentation temperature sensitive) family. FtsH proteases consist of an N-terminal transmembrane domain followed by a hydrophilic region containing an AAA (ATPase associated with various cellular activities) and a protease domain belonging to the M41 peptidase family, which carries a zinc-binding motif and therefore is categorized into the family of zinc metalloproteases^29^,^30^,^31^. Most bacteria have a single gene encoding FtsH, while 12 *FtsH* genes are present in the genome of *Arabidopsis thaliana*. In *Arabidopsis*, three proteins (FtsH3, FtsH4 and FtsH10) are targeted to mitochondria, eight (FtsH1, FtsH2, FtsH5 to FtsH9 and FtsH12) enter the chloroplast, while FtsH11 is dually targeted to both mitochondria and chloroplasts^27^,^28^,^29^,^32^,^33^. In addition, four proteolytically inactive FtsHs (designated as FtsHi) are targeted to chloroplasts^34^. FtsH6 has high-sequence similarity to FtsH2 and FtsH8 (refs 32, 35). Involvement of FtsH6 in degradation of the PSII light harvesting proteins Lhcb1 and Lhcb3 during dark-induced senescence or high-light treatment has been demonstrated *in vitro*^36^, however, additional studies have so far not revealed an *in vivo* function of FtsH6 in high light acclimation or any other biological process^32^,^35^. Notably, *FtsH6* is expressed at very low levels in plants grown at normal growth temperature, but is highly induced after heat treatment in *Arabidopsis* (Arabidopsis eFP Browser; http://bar.utoronto.ca/efp_arabidopsis) and other species across the plant kingdom including the dicot crops rapeseed (*Brassica napus*^37^) and tomato (*Solanum lycopersicum*^38^), and the monocot crops wheat (*Triticum aestivum*^39^) and sorghum (*Sorghum bicolor*^40^). Thus, heat inducibility of *FtsH6* expression appears to be evolutionary conserved in plants suggesting an important role of this metalloprotease in the response to heat stress.

Here we show that the sole plastidic sHSP, HSP21, plays a crucial role in extended thermomemory in *Arabidopsis*. We provide evidence that the ability to maintain high levels of HSP21 protein after priming determines the duration of memory in genetically modified plants as well as natural accessions of *Arabidopsis* with contrasting thermomemory capacity. Furthermore, we show that HSP21 abundance during the memory phase is negatively regulated by heat-induced plastid-localized metalloprotease FtsH6. Our results thus demonstrate the presence of a plastidial FtsH6–HSP21 control module for thermomemory in plants.

## Results

### Identification of thermomemory-associated genes

**Figure 1:**
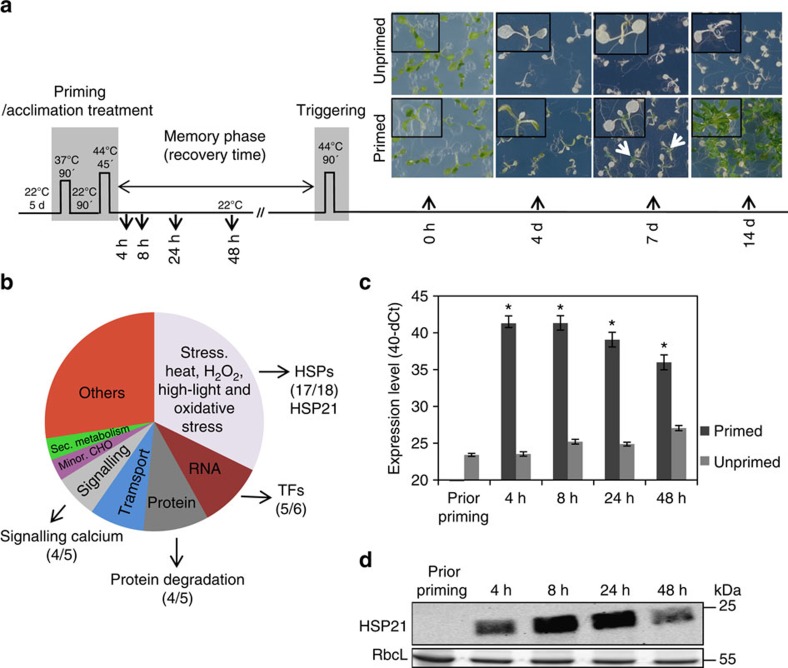
Differential response of primed and unprimed plants to heat stress and identification of thermomemory-associated genes. (**a**) Schematic representation of the thermomemory experimental set-up. Unless otherwise indicated, five-day-old *Arabidopsis thaliana* seedlings were subjected to a priming heat regime of 90 min, 37 °C, followed by 90 min recovery at 22 °C, and 45 min at 44 °C. After priming, seedlings were returned to normal growth condition at 22 °C for 3 or 4 days (memory phase), and then subjected to the heat stress triggering stimulus (90 min, 44 °C). Subsequently, seedlings were transferred to normal growth condition (22 °C) and photographed after 0 h (right after triggering), 4, 7 and 14 days, respectively. Unprimed plants received only the triggering stimulus. Note, cotyledons of both, primed and unprimed seedlings bleach, but only primed plants (not unprimed plants) develop new leaves (arrows point to examples) and continue to grow after triggering. (**b**) Classification of memory-induced genes into functional categories (biological processes) according to their GO term. (**c**) Quantative reverse trancription–PCR and (**d**) Immunoblot analyses revealed enhanced transcript and protein abundance of HSP21 until 48 h into the memory phase in *Arabidopsis* accession Col-0. In **c**, values were expressed as the difference between an arbitrary value of 40 and dCt, so that high 40-dCt value indicates high gene expression level. Error bars indicate means±s.d. of three independent biological replicates each containing a pool of ∼100 seedlings. Asterisks indicate statistically significant difference (*P*<0.01; Student's *t*-test) from the unprimed conditions. In **d**, immunodetection was performed using anti-HSP21 antibody (top panel). RbcL, Ribulose 1,5-bisphosphate carboxylase/oxygenase large subunit (loading control; bottom panel). kDa, kilo Dalton.

To study thermomemory, 5-day-old seedlings of *Arabidopsis* Col-0 plants were subjected to an established thermomemory protocol (see Methods; Fig. 1a). Seedlings of both primed and unprimed plants became pale green and turned white after 4 days of heat stress/triggering stimulus. However, 7 days after the triggering, primed but not unprimed plants started to generate new leaves and grow further (Fig. 1a). To identify genes associated with thermomemory, we performed transcript profiling using Affymetrix ATH1 microarrays comparing Col-0 seedlings 4, 8, 24 and 48 h after the priming stimulus (that is, during the memory phase) with control plants (unprimed). Considering a two-fold cut-off on fold changes (FC), thermomemory-associated genes were selected as follows: genes whose expression was induced 4 h after priming and remained high at all examined time points until 48 h into the memory phase (60 genes), and genes whose expression was downregulated at 4 h after the priming stimulus and remained low until 48 h (19 genes) (Supplementary Data 1).

Upregulated genes were classified into several gene ontology (GO) functional categories, among them ‘response to stress' (that is, high temperature, hydrogen peroxide, high light and oxidative stress) and ‘protein folding' were the most enriched categories (Fig. 1b). Of the 60 upregulated genes, 17 encode for HSPs (Supplementary Data 1). Among the top thermomemory upregulated genes was *HSP21* (*AT4G27670*), which encodes a small HSP (21 kDa monomer size) localized in plastids of both roots and leaves^13^. As shown in Fig. 1c,d, transcript levels of HSP21 as well as its protein abundance remain high until 48 h into the thermomemory phase suggesting a role for this HSP in the maintenance of extended thermomemory in *Arabidopsis*.

### HSP21 confers priming-induced thermotolerance

**Figure 2:**
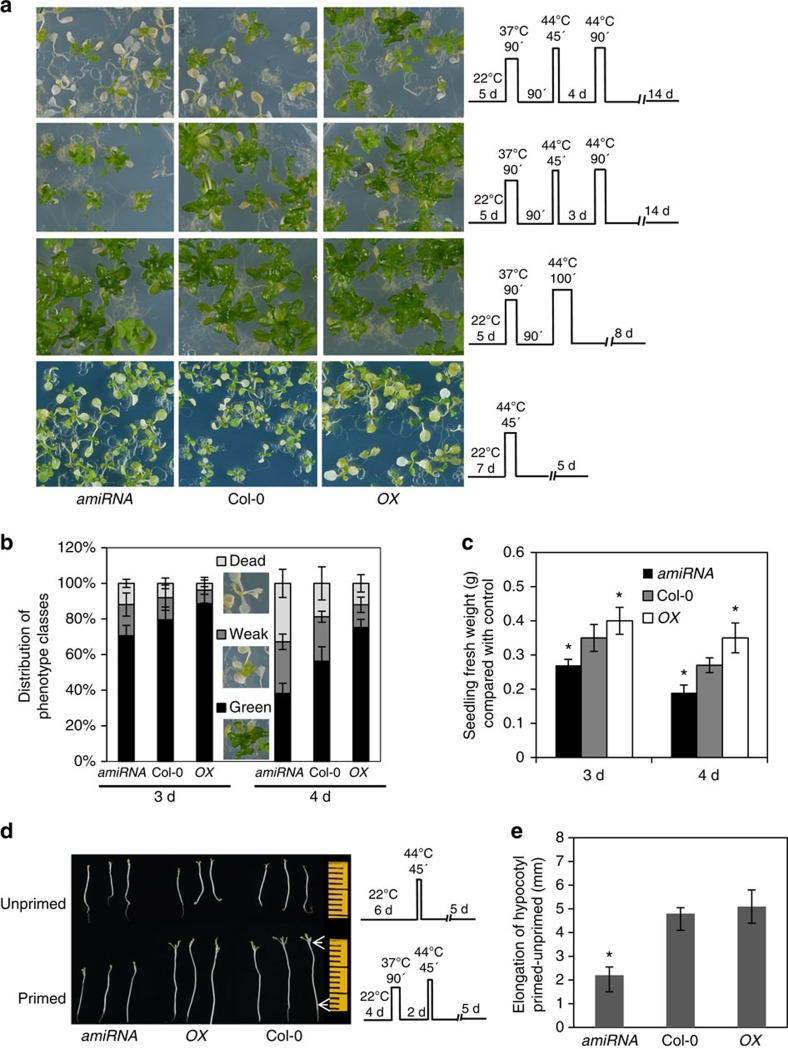
HSP21 affects thermomemory. Seedlings of *HSP21* transgenic and wild-type (Col-0) plants were exposed to different heat stress regimes (schematically shown on the right of **a** and **d**). (**a**) Upper middle and top panels: heat stress triggering after extended recovery, that is, 3 and 4 days, respectively, following the priming treatment. Lower middle panel: acquired heat stress tolerance. Bottom panel: basal heat stress tolerance; heat stress applied to 7-day-old seedlings. Following heat stress, seedlings were transferred to normal growth condition (22 °C) and photographed after 14 days (upper middle and top panels), 8 days (lower middle) or 5 days (bottom). (**b**,**c**) Quantification of results shown in **a** (upper middle and top panels). (**b**) Percentage of seedlings in different phenotype classes. Phenotypes were determined 14 days after triggering. Seedlings were counted as ‘green' (if shoot regeneration was vivid and almost the entire plant was green), ‘weak' (if shoot regeneration was weak and plants were largely pale), or ‘dead'. Representative images are shown. At 3 d recovery, data for *HSP21* transgenic lines are not significantly different from Col-0, except for green seedlings of *HSP21*-amiRNA. At 4-day recovery all data for *HSP21* transgenic lines are different from Col-0, except for ‘weak' seedlings of *HSP21*-amiRNA (*P*<0.05; one-way analysis of variance (ANOVA) least significant difference (LSD) test). (**c**) Seedling fresh weight compared to Col-0. In **b** and **c,** means±s.d. are given (*n*=7 plates with ∼25 seedlings each). (**d**) Hypocotyl elongation of *HSP21* transgenic plants and Col-0 under heat stress. Six-day-old seedlings were subjected to heat stress triggering without (unprimed) or with (primed) pre-treatment at moderate temperature stress as shown. Photos were taken 5 days after triggering. White arrows exemplarily demarcate the upper and lower ends of the hypocotyl. A ruler was included in all images (here shown on the right) to determine hypocotyl lengths. (**e**) Hypocotyl lengths measured 5 days after recovery from the heat stress triggering and compared between primed and unprimed seedlings. Means±s.d. are given (*n*=6 plates with ∼20 seedlings each). amiRNA, *HSP21*-amiRNA line; OX, *35S:HSP21* line. Asterisks in **c** and **e** indicate statistically significant difference (*P*<0.05; one-way ANOVA LSD test) from Col-0.

To investigate whether HSP21 plays a role in heat stress priming and memory, we first generated transgenic *Arabidopsis* lines with enhanced or reduced *HSP21* expression (Supplementary Fig. 1a–d) and subjected 5-day-old *HSP21* transgenic and Col-0 seedlings to our standard priming and triggering stimulus protocol. As shown in Supplementary Fig. 2 there were no significant differences in the development of the *HSP21* transgenic plants and Col-0 before priming as well as after 3 and 4 days of recovery, respectively. *HSP21*-amiRNA and *35S:HSP21* transgenic plants recovered and generated new leaves after a heat stress treatment given 3 days after priming, similar to Col-0 plants (Fig. 2a). Although there was no difference in the recovery rate (Fig. 2b) among *HSP21* transgenics and Col-0 seedlings, the seedling fresh weight was significantly lower in *HSP21*-amiRNA than Col-0 seedlings, but higher in *35S:HSP21* (Fig. 2c). When the memory phase was extended to 4 days, the proportion of highly damaged seedlings (indicated as weak and dead) was significantly increased in Col-0. Notably, *HSP21*-amiRNA plants exhibited a more severe phenotype with significantly less fresh weight than Col-0, while *35S:HSP21* plants showed a higher survival rate and seedling fresh weight under this condition (Fig. 2a, top panel, b, and c). Our data thus clearly indicate that HSP21 is required for *Arabidopsis* plants to retain the memory of a prior exposure to heat stress.

To investigate the specificity of the role of HSP21 for thermomemory, we tested whether altering *HSP21* expression has an effect on basal and acquired heat stress tolerance (see Methods section for details). As shown in Fig. 2a (lower middle and bottom panels), *HSP21* overexpression and knockdown plants were similar in acquired and basal heat stress tolerance to Col-0 control plants suggesting that HSP21 is specifically required for maintenance of thermomemory.

Hypocotyl elongation assays have previously been employed for testing the effect of temperature on seedling growth^41^,^42^. Therefore, we next analysed hypocotyl growth in the *HSP21* transgenic plants to test for changes in the thermomemory. To this end 4-day-old etiolated seedlings were subjected to the following temperature regime: 1.5 h, 37 °C (priming stimulus); 2 days recovery at 22 °C; and 45 min, 44 °C (triggering stimulus). As control, 6-day-old etiolated seedlings were subjected to 45 min, 44 °C without pre-adaptation. Hypocotyl elongation was quantified five days after the triggering stimulus. As shown in Fig. 2d, all genotypes failed to elongate their hypocotyl after treatment at 44 °C when no pre-treatment was given. However, wild-type and *HSP21* overexpression seedlings retained the ability for hypocotyl elongation when 44 °C was given 2 days after a priming stimulus. In contrast, *HSP21*-amiRNA seedlings showed a severe block of hypocotyl elongation under this condition (Fig. 2d,e) further supporting the model that HSP21 is crucial for extended memory maintenance.

### HSP21 is more abundant in strong memory accession N13

**Figure 3:**
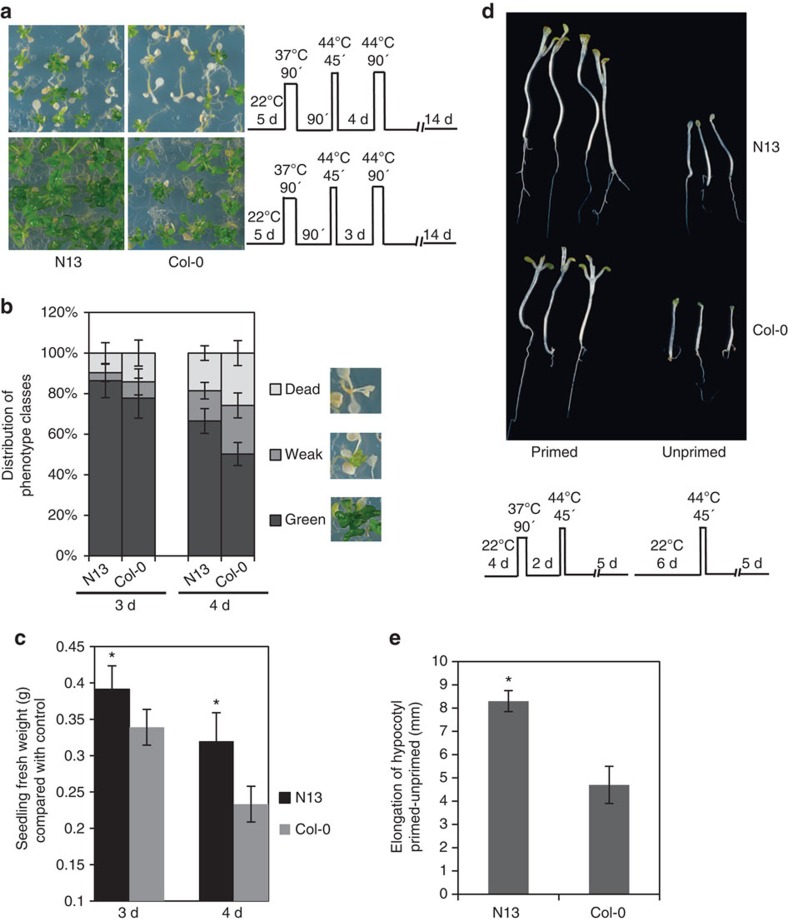
Accession N13 has a superior ability to memorise a past heat stress. (**a**) Five-day-old seedlings of *Arabidopsis* accessions N13 and Col-0 were subjected to the heat stress triggering stimulus 3 or 4 days after priming, as shown schematically on the right of each section. (**b**,**c**) Quantification of phenotypes. (**b**) Percentage of seedlings in different phenotype classes. Images show representative phenotype categories as explained in legend to Fig. 2b. At 3 d recovery, data for N13 are not significantly different from Col-0. At 4 d recovery all data for N13 are different from Col-0 (*P*<0.05; Student's *t*-test). (**c**) Seedling fresh weight compared to control plants. In **b** and **c,** means±s.d. are given (*n*=10 plates with ∼25 seedlings each). (**d**) Hypocotyl elongation after heat stress. Six-day-old seedlings were subjected to the heat stress triggering stimulus without (unprimed) or with (primed) pre-treatment with moderate temperature stress as shown schematically. Photos were taken 5 days after triggering. (**e**) Hypocotyl lengths measured 5 days after recovery from the heat stress triggering stimulus and compared between primed and unprimed conditions. Means±s.d. are given (*n*=6 plates with ∼20 seedlings each). Asterisks in **c** and **e** indicate statistically significant difference (*P*<0.01; Student's *t*-test) from the Col-0 control.

Accessions of *Arabidopsis* offer an excellent tool to explore natural diversification of a biological process of interest. Thus, to identify an accession with a better thermomemory than Col-0 we performed a screen (performed twice) with 40 additional *Arabidopsis* accessions (see Methods section) for differences in heat stress priming and memory. We identified N13 as a strong memory accession, which survives high-temperature stress (90 min, 44 °C), given 3 or 4 days after the priming treatment, significantly better than the Col-0 control. As displayed in Fig. 3a, both Col-0 and N13 survived heat stress applied 3 days after the priming treatment; the fresh weight of seedlings was significantly lower in Col-0 compared to N13 when analysed 14 days after the heat stress triggering stimulus. When 4 days was applied as the memory time, we observed a higher number of damaged seedlings for Col-0, whereas N13 seedlings recovered significantly better from severe repeated heat stress (Fig. 3a–c) demonstrating that N13 was better able to respond to the heat priming stimulus and to memorise the past heat stress experience. This conclusion was supported by analysing hypocotyl elongation in N13 and Col-0 under the condition of heat stress. Hypocotyl elongation after the triggering heat stress was more prominent in N13 than Col-0 (Fig. 3d,e). We did not observe any significant difference between N13 and Col-0 seedlings for basal heat stress tolerance, when 7-day-old seedlings were subjected to 45 min, 44 °C (Supplementary Fig. 3).

**Figure 4:**
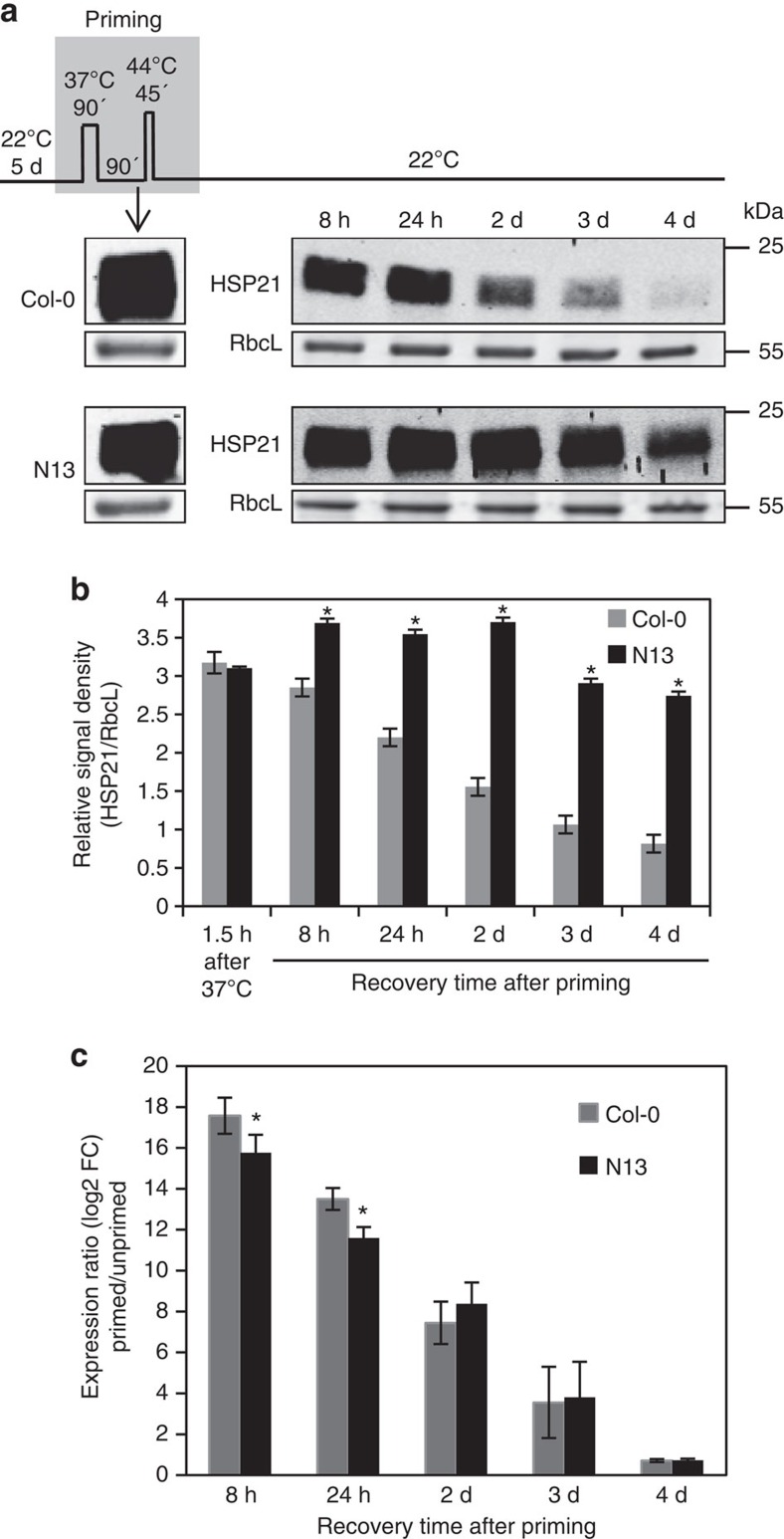
Differential abundance of HSP21 in *Arabidopsis* accessions N13 and Col-0 during the thermomemory phase. (**a**) Immunoblot analysis of HSP21 protein level after the first step of priming treatment (90 min, 37 °C) and during the thermomemory phase. RbcL, Ribulose 1,5-bisphosphate carboxylase/oxygenase large subunit (loading control). kDa, kilo Dalton. (**b**) Signals of immunoblot analyses were quantified using ImageJ and normalized to the amount of RbcL in the same samples. Means±s.d. are given (*n*=3, independent biological replicates each representing a pool of ∼120 seedlings grown on six plates; full gel blots used for the quantifications are shown in Supplementary Fig. 10). (**c**) *HSP21* expression in N13 and Col-0 seedlings during memory phase compared to unprimed controls. FC, fold change. Means±s.d. (*n*=3, independent biological replicates each representing a pool of ∼120 seedlings grown on six plates). Asterisks in **b** and **c** indicate statistically significant difference (*P*<0.05; Student's *t*-test) from the Col-0 control.

To investigate whether variation in HSP21 level contributes to the differential thermomemory performance of N13 and Col-0, we analysed its transcript and protein abundance in the two accessions at different time points along the memory phase. HSP21 protein was undetectable in both, Col-0 and N13 in unprimed conditions, but as seen in Fig. 4a, pronounced accumulation of HSP21 to the same level was evident in both accessions already 1.5 h after the first phase of the priming stimulus (1.5 h, 37 °C). However, during the thermomemory phase, HSP21 protein level progressively decreased to about 25% of its heat stress-induced level in Col-0 by day 4, but remained at above 80% of its induced level in N13 (Fig. 4a,b). At day 4, HSP21 protein was 3.4-fold more abundant in N13 than in Col-0 (Fig. 4b). Our data thus support a model where HSP21 plays an important role in the molecular machinery of thermomemory.

We then compared *HSP21* expression in Col-0 and N13 during the memory phase. *HSP21* was significantly higher expressed in Col-0 than N13 at 8 h and 24 h after the priming, but this difference vanished at the later time points (2, 3 and 4 days), suggesting that the diminished HSP21 protein in Col-0 is not correlated with *HSP21* transcript level (Fig. 4c).

To investigate whether the more rapid decrease of HSP21 protein level in Col-0 compared to N13 during the thermomemory phase is due to an amino acid sequence polymorphism(s), which might alter protein structure and/or stability, we compared the amino acid sequences of HSP21 from the accessions. To strengthen our analysis, we included one more accession, Tsu-0, with a similar pattern of HSP21 accumulation and thermomemory behaviour as Col-0 (Supplementary Fig. 4). Sequence analysis of complementary DNA (cDNA) from the selected accessions identified one polymorphic site as amino acid codon 77, which in Col-0 encodes for threonine (ACC) but for alanine (GCC) in both, N13 and Tsu-0 (Supplementary Fig. 4a and b). Furthermore, *HSP21* transcript levels during the recovery/memory phase were similar in the three examined accessions (Supplementary Fig. 4c). Therefore, we concluded that the amino acid polymorphism in the N13 protein sequence is unlikely to be responsible for higher accumulation/stability of HSP21 and consequently strong thermomemory.

Taken together, the results suggest that HSP21 protein abundance during the thermomemory is controlled by regulatory mechanisms at the translational and/or post-translation level.

### Cycloheximide blocks decline of HSP21 in Col-0 during memory

**Figure 5:**
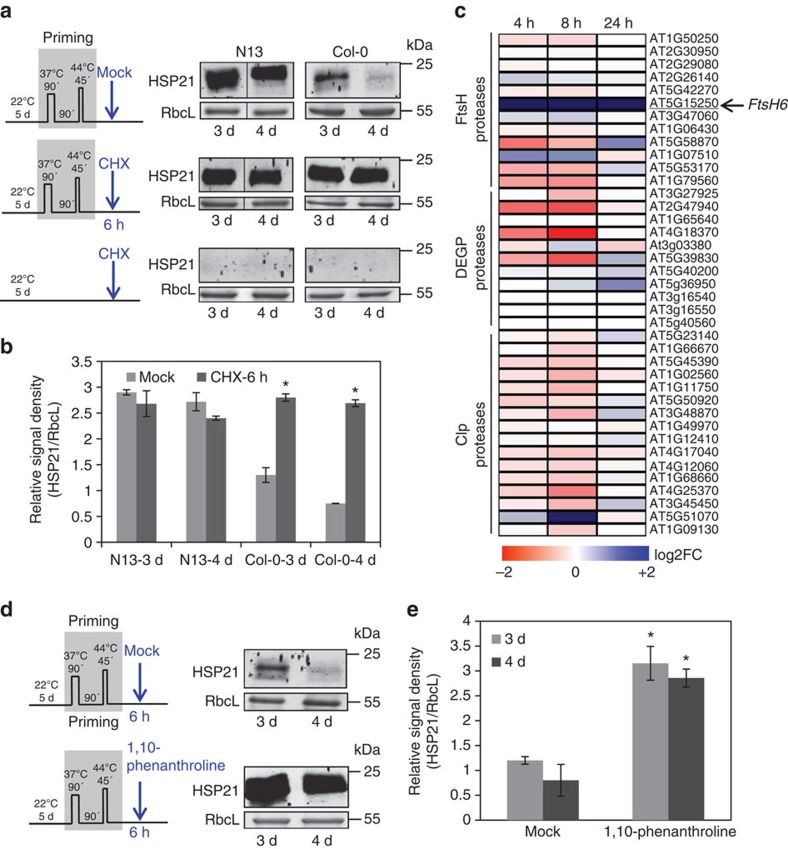
The effect of cycloheximide and 1,10-phenanthroline on the accumulation of HSP21. (**a**) Immunoblot analyses of HSP21 in accessions N13 and Col-0 at days 3 and 4 of the memory phase on cycloheximide (CHX) and mock (0.1% dimethylsulphoxide) treatments. CHX or mock treatment was applied to *Arabidopsis* seedlings at 6 h into the memory phase, as shown schematically on the left of each section. Bottom panel: Immunoblot analyses of HSP21 in accessions N13 and Col-0 in unprimed samples. Note the absence of HSP21 protein. RbcL, Ribulose 1,5-bisphosphate carboxylase/oxygenase, large subunit (loading control). Bands shown for mock- and CHX-treated N13 seedlings (primed) have been rearranged for presentation purpose. The original blot images are shown in Supplementary Fig. 10. (**b**) Signals of immunoblot analyses like in **a** were quantified using ImageJ and normalized to the amount of RbcL in the same samples. Mean±s.d. are given (*n*=3, independent biological replicates each representing a pool of ∼100 seedlings grown on five plates). Asterisks indicate significant difference in the level of HSP21 between CHX- and mock-treated samples at each indicated time point (*P*<0.05; Student's *t*-test). (**c**) Heat map showing the fold change (log2 basis) of the expression of major nuclear-encoded (plastid) chloroplast proteases in Col-0 seedlings at 4 h, 8 h and 24 h after the priming stimulus (that is, during the memory phase) compared with control plants (unprimed). Blue, upregulated; red, downregulated; scale bar given at the bottom of the heat map. (**d**) Immunoblot analyses of HSP21 protein abundance in Col-0 seedlings at days 3 and 4 days of the memory phase on treatment with 1,10-phenanthroline (a metalloprotease inhibitor). Seedlings were harvested for immunoblotting after days 3 or 4. Immunodetection was performed using anti-HSP21 antibody. RbcL, Ribulose 1,5-bisphosphate carboxylase/oxygenase large subunit (loading control). (**e**) Signals of immunoblot analyses like in **d** were quantified using ImageJ and normalized to the amount of RbcL in the same samples. Means±s.d. (*n*=3, independent biological replicates each representing a pool of ∼100 seedlings grown on five plates). Asterisks indicate significant difference in the level of HSP21 between mock- and 1,10-phenanthroline-treated samples at each indicated time point (*P*<0.05; Student's *t*-test). kDa, kilo Dalton. Uncropped gel blots used for the quantification of data in **b** and **e** are shown in Supplementary Fig. 10.

To test whether high levels of HSP21 protein at later memory time points in accession N13 require *de novo* protein synthesis, we used cycloheximide (CHX) to inhibit protein translation. To this end, seedlings of both Col-0 and N13 were treated with CHX at 6 h after priming (when HSP21 level was high; Fig. 4a,b), and HSP21 protein level was analysed by immunoblotting at days 3 and 4 into the memory phase. As shown in Fig. 5a,b, CHX treatment did not inhibit the induction of HSP21 in N13 revealing that *de novo* translation is not the primary cause for accumulation of HSP21 in N13 at days 3 and 4 of the memory phase. Interestingly, CHX treatment of Col-0 seedlings resulted in significantly higher accumulation of HSP21, more evident at day 4 of the memory phase, indicating the presence of an unknown protein in Col-0, possibly a protease, whose *de novo* translation is required for HSP21 degradation at a later stage of the memory phase.

### FtsH6 metalloprotease affects HSP21 level during memory

In an attempt to understand how HSP21 is degraded at the later stage of the memory phase, we first examined our thermomemory Affymetrix transcriptome data for expression of major nuclear-encoded (plastid) chloroplast proteases including stromal ATP-dependent Clp proteases, lumenal DegPs and the FtsH metalloproteases^27^,^29^. Interestingly, of 39 genes for plastid proteases the expression of only 1 gene encoding an FtsH metalloprotease (AT5G15250; FtsH6) was enhanced at 4 h into the memory phase and remained high until 24 h (Fig. 5c and Supplementary Data 2). FtsH6 (filamentation temperature-sensitive H6) is a nuclear-encoded chloroplast zinc- and ATP-dependent protease associated with the thylakoid membrane. Expression of *FtsH6* is strongly induced by heat in *Arabidopsis* and other plant species including rapeseed^37^, wheat^39^, sorghum^40^ and tomato^38^.

To test a potential involvement of metalloproteases for HSP21 degradation, we treated Col-0 seedlings with the metalloprotease inhibitor 1,10-phenanthroline and detected HSP21 protein by immunoblotting at days 3 and 4 of the memory phase. As shown in Fig. 5d,e, treatment with 1,10-phenanthroline largely blocked the late thermomemory decrease of HSP21 protein level. The sustained induction of *FtsH6* expression during the thermomemory phase as well as inhibition of HSP21 degradation on treatment with 1,10-phenanthroline suggest FtsH6 as a candidate protease involved in the degradation of HSP21 during the memory phase in Col-0.

**Figure 6:**
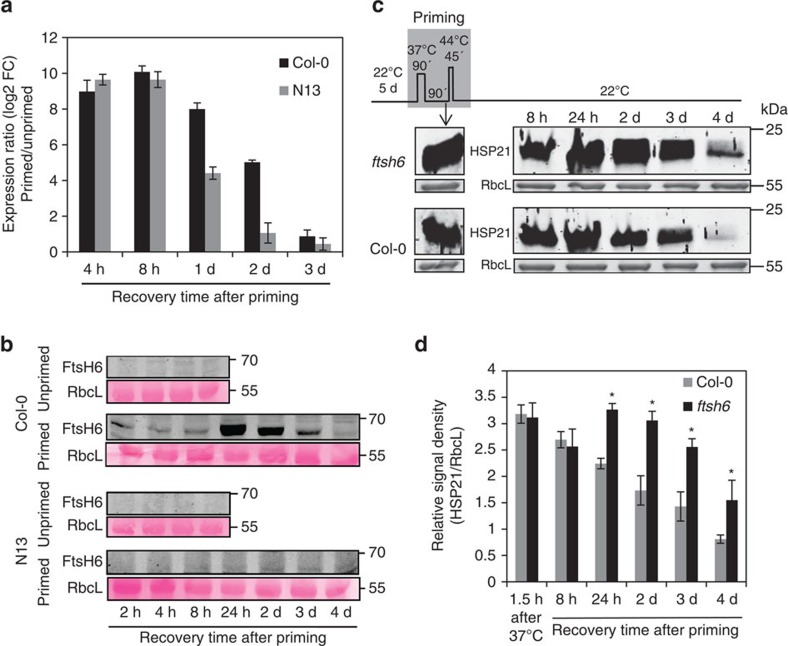
Metalloprotease FtsH6 is involved in thermopriming. (**a**) *FtsH6* expression in *Arabidopsis* N13 and Col-0 seedlings during the memory phase compared to unprimed controls. FC, fold change. Error bars indicate means±s.d. of three independent biological replicates each containing a pool of ∼160 seedlings. (**b**) Immunoblot analysis of FtsH6 protein in accessions Col-0 and N13 at different time points of the memory phase. Immunodetection was performed using anti-FtsH6 antibody (top panels). RbcL, Ribulose 1,5-bisphosphate carboxylase/oxygenase large subunit (loading control; bottom panels). Note the absence of FtsH6 protein in Col-0 unprimed samples and in N13 unprimed and primed samples. (**c**) Immunoblot analysis of HSP21 protein in *ftsh6* mutant (*Salk_012429*) and Col-0 seedlings after the first step of the priming treatment (90 min, 37 °C) and during the thermomemory phase. Immunodetection was performed using anti-HSP21 antibody (top panel). RbcL, ribulose 1,5-bisphosphate carboxylase/oxygenase large subunit (loading control; bottom panel). (**d**) Signals of the immunoblot analyses were quantified using ImageJ and normalized to the amount of RbcL in the same samples. Means±s.d. are given (*n*=3, independent biological replicates each representing a pool of ∼100 seedlings grown on five plates; gel blots used for the quantification are shown in Supplementary Fig. 10). Asterisks indicate statistically significant difference (*P*<0.05; Student's *t*-test) from the Col-0 control. kDa, kilo Dalton.

To investigate whether variation in FtsH6 is responsible for differential abundance of HSP21 protein in N13 and Col-0, and thus their contrasting thermomemory behaviour, we first compared expression of *FtsH6* between Col-0 and N13 during the memory phase (until 3 days) by quantative reverse trancription–PCR (Fig. 6a). In Col-0, the expression of *FtsH*6 was drastically induced on priming treatment and remained high until day 2 of the memory phase. We observed a similar induction pattern for *FtsH6* in N13, albeit with a more rapid decline at the later time points. However, as we show below, N13 *FtsH6* transcript is non-functional.

Next we detected FtsH6 protein by immunoblot analysis in N13 and Col-0 seedlings at different time points of the memory phase and compared it with control plants (unprimed). As shown in Fig. 6b, FtsH6 was undetectable in both Col-0 and N13 under unprimed condition. In Col-0, FtsH6 protein was abundant until day 3 of the memory phase in accordance with its higher transcript levels. Intriguingly, however, no FtsH6 protein was detected in N13 during the entire memory phase, even at an earlier time point (4 h) when its transcript was still abundant. Comparing the FtsH6 coding sequences of N13 and Col-0, we identified several nucleotide polymorphisms (Supplementary Fig. 5a), including a single-nucleotide deletion (nucleotide ‘G' at position 379 of the Col-0 coding sequence is deleted in N13) resulting in a frame-shift mutation and a premature stop codon in the N13 sequence. This observation explains the absence of FtsH6 protein in N13. Only a few amino acid substitutions (0 to 3 residues) were found in FtsH6 proteins of the 30 other accessions we tested for heat stress priming and triggering compared with Col-0 (see above; Supplementary Fig. 5b); no genome sequences were available for the remaining 9 accessions.

This finding provides further evidence for FtsH6 as a potential protease involved in the regulation of HSP21 turnover during thermomemory. Lack of functional FtsH6 in N13 could be responsible for the higher accumulation of HSP21 and therefore enhanced thermomemory in this accession.

If FtsH6 is a key protease for degradation of HSP21 during the memory phase, the decrease in HSP21 level at later stage of thermomemory phase should be retarded in an *ftsh6* knockout mutant. Therefore, we compared HSP21 protein level of wild type and the *ftsh6* mutant (Salk_012429; Col-0 background) at different stages of thermomemory phase. As shown in Fig. 6c,d, protein level of HSP21 was identical in both genotypes after the first step of priming treatment (1.5 h, 37 °C) and until 8 h of the thermomemory phase. However, at later stages (24 h, 2, 3 and 4 days) the level of HSP21 was significantly higher in the *ftsh6* mutant than in Col-0 (about 1.8-fold higher in *ftsh6* than Col-0 at days 2, 3 and 4; Fig. 6d). Our data thus show that FtsH6 is involved in the degradation of HSP21 at the later stages of the memory phase. A slightly higher accumulation of HSP21 protein was observed after 1,10-phenanthroline treatment (Fig. 5d,e) compared to the *ftsh6* mutant, suggesting that other metalloproteases are also involved in controlling the level of HSP21 later in the memory phase.

We next subjected 5-day-old *ftsh6* mutant and Col-0 seedlings to our priming and triggering stimulus protocol. *ftsh6* mutants revealed a slightly better thermomemory behaviour than Col-0, measured by seedling survival rate and fresh weight (Supplementary Fig. 6a–c). However, *ftsh6* plants had similar basal and acquired heat stress tolerance as Col-0 control plants. We also determined the role of FtsH6 for thermomemory by analysing hypocotyl elongation after priming and triggering and observed that hypocotyl elongation was much less affected by the triggering heat stress in the *ftsh6* mutant than the wild type (Col-0; Supplementary Fig. 6d and e), underscoring the contribution of FtsH6 to restricting the thermomemory by controlling HSP21 abundance.

To test whether the effect of CHX treatment on the induction of HSP21 protein level in the Col-0 accession (Fig. 5a,b) is due to the inhibition of *de novo* synthesis of FtsH6, Col-0 seedlings were treated with CHX at 6 h after priming and FtsH6 protein level was analysed by immunoblotting at days 2, 3 and 4 into the memory phase. As illustrated in Supplementary Figure 7a and b, treatment with CHX resulted in a significant reduction of the level of FtsH6 during thermomemory phase. This experiment shows that degradation of HSP21 during the memory phase in Col-0 correlates with enhanced FtsH6 protein synthesis.

**Figure 7:**
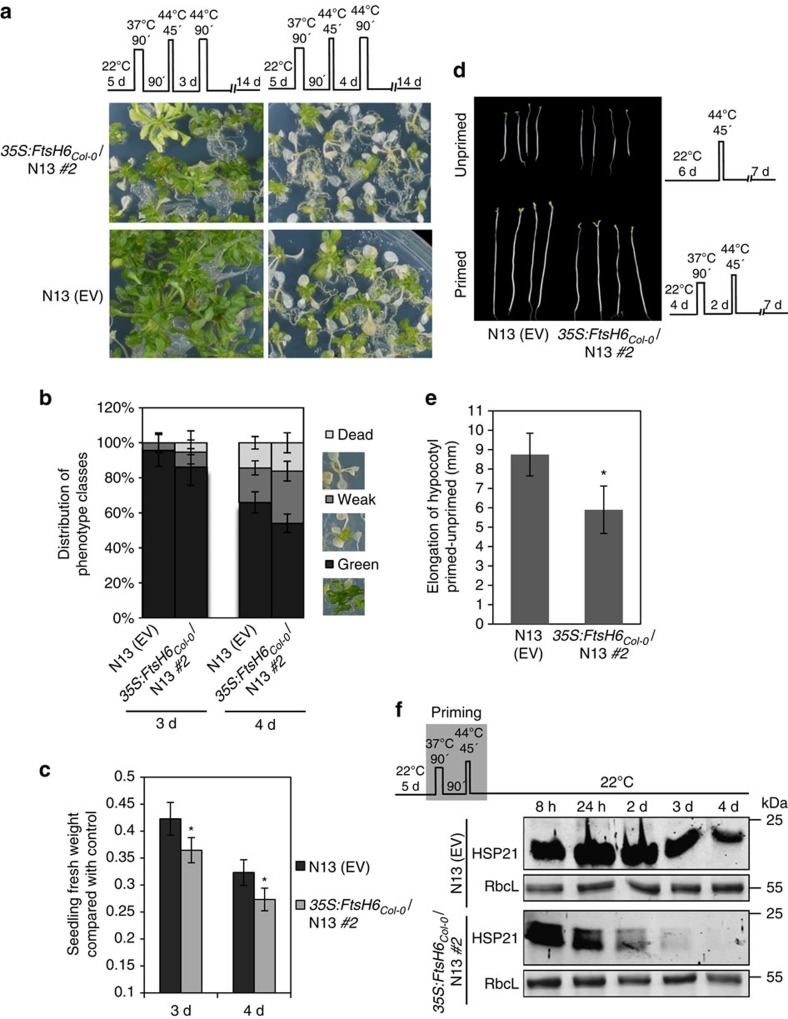
Overexpression of *FtsH6* restricts thermomemory in N13. (**a**) Seedlings of *35S:FtsH6*_*Col-0*_/N13 and N13 EV were exposed to different heat stress regimes schematically shown on the top of each section; heat stress triggering stimulus after 3 days (left panels) and 4 days (right panels), respectively, following the priming treatment. (**b**,**c**) Quantification of results shown in **a**. (**b**) The percentage of seedlings in different phenotype classes. Phenotype analysis was performed 14 days after the triggering stimulus. Images show representative phenotype categories as explained in legend to Fig. 2b. At 3 d and 4 d recovery, data for *35S:FtsH6*_*Col-0*_/N13 are significantly different from N13, except for dead seedlings of *35S:FtsH6*_*Col-0*_/N13 at 4 days (*P*<0.05; Student's *t*-test). (**c**) Seedling fresh weight compared to control plants. Means±s.d. are given (*n*=10 plates with ∼25 seedlings each). (**d**) Hypocotyl elongation assay for *35S:FtsH6*_*Col-0*_/N13 and N13 EV seedlings under heat stress. Six-day-old seedlings were subjected to heat stress triggering stimulus without (unprimed) or with (primed) pre-treatment with moderate temperature stress as shown schematically. Photos were taken 7 days after triggering. (**e**) Hypocotyl lengths measured 7 days after recovery from the heat stress triggering stimulus and compared between primed and unprimed seedlings. Means±s.d. are given (*n*=10 plates with ∼20 seedlings each). Asterisks in **c** and **e** indicate statistically significant difference (*P*<0.05; Student's *t*-test) from the N13 EV control. (**f**) Immunoblot analysis of HSP21 protein in *35S:FtsH6*_*Col-0*_/N13 and N13 EV seedlings during the thermomemory phase. Immunodetection was performed using anti-HSP21 antibody (top panel). RbcL, ribulose 1,5-bisphosphate carboxylase/oxygenase large subunit (loading control; bottom panels). kDa, kilo Dalton.

Finally, we overexpressed *FtsH6* from Col-0 in the N13 accession (*35S:FtsH6*_*Col-0*_/N13 plants; Supplementary Fig. 8a and b) and tested the effect of this on the thermomemory. Both, the seedling survival assay (Fig. 7a–c) and the hypocotyl elongation assay (Fig. 7d,e) revealed a decreased thermomemory on *FtsH6* overexpression in the N13 accession. Furthermore, HSP21 protein level declined much faster in the *35S:FtsH6*_*Col-0*_/N13 than in N13 empty-vector (EV) control plants during the thermomemory phase (Fig. 7f), supporting our conclusion that FtsH6 plays a key role in determining HSP21 abundance during memory. In accordance, we did not detect a difference between *35S:FtsH6*_*Col-0*_/N13 overexpressors and control plants when testing basal and acquired heat stress tolerance (Supplementary Fig. 8c).

## Discussion

Although the importance of stress priming and memory in bacteria and plants is increasingly recognized^1^,^2^, the details of the molecular mechanisms underlying the stress memory phenomenon remain largely unexplored, in particular in plants. Experimental evidence indicates that plants have an ‘epigenetic' memory, which may involve changes at the chromatin level including histone modifications and DNA methylation, which both occur in the response to stress^43^,^44^,^45^,^46^,^47^. Another mechanism for stress priming in plants could be RNA polymerase II stalling at gene promoters, which was suggested as a memory mark after drought stress in *Arabidopsis*^48^. The accumulation of inactive transcription factors (or cofactors) after a priming stimulus, and their activation on experiencing the triggering stimulus, may represent a further molecular mechanism of priming and memory^49^,^50^,^51^. Currently, however, details about how transcription factors affect thermomemory in plants are largely missing, although HsfA2 has been identified as a key element in this^25^. HsfA2 regulates the expression of a number of *HSP* genes^52^,^53^ and in a transcription regulatory cascade is located downstream of the NAC transcription factor JUNGBRUNNEN1 (JUB1), which like *HSFA2* shows high expression during the thermomemory phase^54^,^55^. *Hsa32* encoding a heat shock-associated protein of 32 kDa has also been shown to be a key component of thermomemory in *Arabidopsis* by interacting with HSP101 (refs 41, 56). In addition, microRNAs (miRNAs), which cause the degradation of mRNAs or translational inhibition, contribute to modulating the priming of stress responses in plants^57^. Finally, priming and stress memory might involve metabolic changes that are maintained throughout the memory phase, thus allowing a more rapid response of the plant to an upcoming new stress^58^,^59^,^60^.

**Figure 8:**
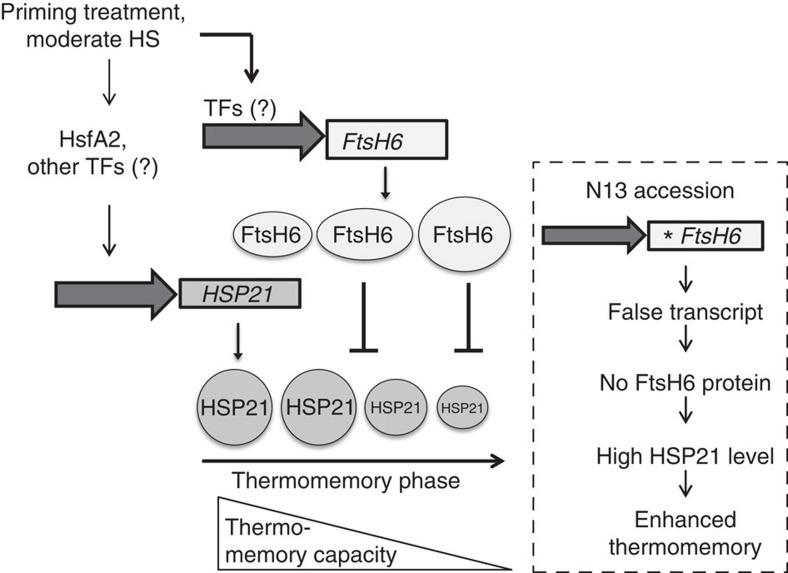
Proposed model for the regulation of thermomemory via the control of HSP21 protein abundance by FtsH6. A priming treatment (heat stress) induces *HSP21* and *FtsH6* expression, and accumulation of the two plastidial proteins. On progression into the memory phase, HSP21 protein abundance decreases due to FstH6 activity, which restricts the duration of the thermomemory. Right: No FtsH6 protein is produced in *Arabidopsis* accession N13, due to DNA polymorphisms in its coding sequence (the asterisk indicates the premature stop codon in the N13 *FtsH6* sequence). The lack of FtsH6 allows HSP21 protein to remain at higher abundance for a longer time, thereby extending thermomemory duration. In addition to HSP21, FtsH6 may control the abundance of other, currently unknown, target proteins during the thermomemory phase (not indicated).

Our results presented here provide evidence for a role of the plastid-localized sHSP HSP21–FtsH6 control module for thermomemory, whereby HSP21 protein abundance is negatively controlled by heat stress-induced FtsH6 (see model in Fig. 8). Previous studies have indicated a role of HSP21 in protecting photosystem II against oxidative and heat stress, a function that may be achieved by HSP21's ability to maintain chloroplast function through an interaction with pTAC5, a nucleoid protein that interacts with the plastid-encoded RNA polymerase-dependent transcription complex, an important component of the chloroplast transcription machinery^19^,^21^. This capacity of HSP21 might be key to the role it has for extending the thermomemory. Notably, the abundance of cytosolic HSP101 is not affected during the thermomemory phase when *FtsH6* is mutated (Supplementary Fig. 9), suggesting a specific role of FtsH6 for the plastidic component of plant thermomemory.

Importantly, we discovered genetic diversity of thermomemory-related HSP21 accumulation in two natural accessions of *Arabidopsis* contrasting in their thermomemory capacity. HSP21 protein abundance remained elevated for longer in accession N13, which we show has strong thermomemory, than in Col-0, which has a weak thermomemory. Using a combined pharmacological (protease inhibitor) and transcriptomic approach, we identified plastid-localized metalloprotease encoded by *FtsH6* to be functionally involved in regulating the abundance of HSP21, in particular towards the end of the thermomemory phase. Notably, *FtsH6* is mutated in N13, precluding the formation of FtsH6 protein, as confirmed by western blot analysis, while in Col-0, FtsH6 protein is readily detected after heat stress priming (Fig. 6b). Expressing *FstH6* from Col-0 in N13 leads to a faster decline of HSP21 protein abundance during the thermomemory phase, concomitant with a reduced thermomemory compared with the N13 control line (Fig. 7), providing further evidence for the important role of FtsH6 in determining the thermomemory (Fig. 8).

A biological function for FtsH6 has not been identified previously and *ftsh6* knockout mutants did not show a detectable phenotype under the experimental conditions applied earlier^35^,^36^, while FtsH1, FtsH2, FtsH5 and FtsH8 are involved in the degradation of photodamaged D1 protein of photosystem II, together with lumenal Deg proteases (reviewed in Wagner *et al*.^29^). In contrast to other *FtsH* genes, *FtsH6* is rapidly and highly induced by heat stress in *Arabidopsis* (Fig. 5c) and other species including dicot and monocot crops^37^,^38^,^39^,^40^, indicating evolutionary conservation and ecological relevance of *FtsH6* heat inducibility. In accordance with this, our data presented here demonstrate a role of FtsH6 in regulating the stability of HSP21 after a priming heat stress, although the molecular mechanism through which this occurs is not clear at present. Whether FtsH6 has a similar function in other plant species, including crops, needs to be determined in the future.

Although FtsH6 plays an important role in regulating HSP21 protein level it is likely not the only metalloprotease involved, as indicated by the fact that 1,10-phenanthroline treatment triggers a higher accumulation of HSP21 than knocking out *FtsH6* (*cf.* Figs 5e and 6d). This finding is in accordance with the observation that *ftsh6* has only a mild thermomemory effect in the survival assay, albeit a much stronger effect is observed when the mutant is tested in the hypocotyl elongation assay (Supplementary Fig. 6d and e). Finding further molecular processes controlling HSP21 protein level during the thermomemory phase will therefore remain an important task for the future.

Both *HSP21* and *FtsH6* are strongly induced by heat stress. *HSP21* is a target of HsfA2 (ref. 52), a heat-inducible transcription factor that plays a crucial role for setting the thermomemory in *Arabidopsis*^25^. It is also upregulated by HsfA1a/b^61^, indicating that heat-induced expression of *HSP21* is under the control of multiple heat-associated transcription factors. However, a direct upstream regulator of *FtsH6* has not been reported so far, suggesting that a currently unknown upstream transcription factor(s) controls expression of *FtsH6* during heat stress. We thus propose that coordinated expressional regulation of the *HSP21* and *FtsH6* genes by different transcription factors contributes to the fine-tuning of the thermomemory. Future research has to uncover the details of this transcriptional regulatory network that underlies heat stress priming and that helps the plant to configure its thermomemory.

In conclusion, our work demonstrates a crucial role of chloroplasts (or plastids in general) for the duration of the thermomemory in plants, which involves the plastid sHSP HSP21 in combination with FtsH6 metalloprotease as central components: continued high levels of HSP21 protein abundance after a priming heat stress promote the extension of the memory phase (Fig. 8). The level of HSP21 at the later phase of the thermomemory is negatively controlled by FtsH6, which is missing in the strong thermomemory accession N13, but produced on heat stress in the weak thermomemory accession Col-0. Besides HSP21, FtsH6 may interact with other proteins to determine the extent of the thermomemory, but such further targets remain unknown at present. The analysis of the proteome of *FtsH6*-modified lines (knockouts or overexpressors) at different time points throughout the memory phase may be a fruitful approach to identify additional targets of FtsH6. Furthermore, as natural variation exists for the HSP21–FtsH6 control module, addressing the selective forces that maintain this variation, as well as possible trade-offs that might be associated with an extended thermomemory, are further interesting aspects to study in the future. In addition, considering the involvement of plastids in establishing the thermomemory it is an interesting observation that chloroplast translation involving chloroplast ribosomal protein S1 is required for the heat stress-induced expression of *HsfA2*; however, whether ribosomal protein S1 is also required for thermomemory was not tested^62^.

Regulating the abundance of chaperones such as HSP21 after a priming heat stress could be a general mechanism for setting the temporal frame for maintaining thermomemory (or the memory to any other type of stress). Similarly, Hsa32 and large HSP HSP101 have recently been shown to form a regulatory interaction circuit in the cytosol that controls the abundance of HSP101 by Hsa32 and thereby thermomemory^56^. We propose that the control of HSP (or, more general, chaperone protein) abundance is a key mechanism for determining the duration of the memory phase. Future research will unravel the molecular details of the cellular networks that control the abundance and functionality of heat stress-related chaperones as key components of the plant's thermomemory.

## Methods

### Plant material and growth conditions

The following *Arabidopsis thaliana* (L.) Heynh. accessions were included in our preliminary screen for differences in thermomemory: Col-0, N13, Cvi-0, C24, Ct, Can-0, Lip-0, Mdn-1, Mh-0, Mt-0, Aitba-1, Abd-0, Bl-1, Bur-0, Dog-4, Tsu-0, St-0, Kas-1, Yo-0, Xan-1, Kondara, Ler-0, Ko-2, Ri-0, Fei-0, Leo-1, Yeg-1, Ste-0, ICE1, Kin-0, Le-0, Oy-0, N14, Sah-0, Berk, Hiro, Ita, HEK2, Stepn, Shign and Borsk2. Seeds were obtained from the *Arabidopsis thaliana* Resource Centre for Genomics (INRA, France; http://dbsgap.versailles.inra.fr/publiclines), or from Ellen Zuther and Roosa Laitinen, Max Planck Institute of Molecular Plant Physiology, Potsdam, Germany. Surface-sterilized seeds were germinated in Petri dishes containing an identical volume of Murashige-Skoog agar medium supplemented with 1% sucrose (w/v)) and seedlings were grown under a diurnal cycle of 16 h light (120 μE m^−2^ s^−1^) at 22 °C and 8 h dark at 22 °C.

### Generation of transgenic plants

Constructs were generated by PCR and restriction enzyme-mediated cloning. Sequences of oligonucleotides (Eurofins MWG Operon; Ebersberg, Germany) are given in Supplementary Table 1. PCR-generated amplicons were checked by DNA sequence analysis (MWG or Seqlab). To generate *HSP21* knockdown plants we engineered amiRNAs by replacing the original *miR319a*/miR319a* sequence in plasmid pRS300 (ref. 63) with *HSP21*-specific amiRNA sequences. To this end, three different amiRNAs (*amiRNA1*: 5′-TTAGTATCTAACATTTGTCGC-3′; *amiRNA2*: 5′-TGTATCTAACATTTGTCGCAT-3′; *amiRNA3*: 5′-TATAATGTTGATCGAGTCCTA-3′) were designed using ‘WMD3: Web MicroRNA Designer 3' to target different sites of the *HSP21* transcript (Supplementary Fig. 1). The amiRNA constructs were subsequently cloned via added *Pme*I*-Pac*I sites into pGreen0229-35S plant transformation vector^64^ behind the cauliflower mosaic virus (CaMV) 35S promoter. For *35S:HSP21*, the *HSP21* open reading frame was amplified by PCR from *Arabidopsis* Col-0 leaf cDNA, inserted into pCR2.1 vector using TA cloning kit (Invitrogen), and then cloned *via* added *Pme*I*–Pac*I sites into pGreen0229-35S. The *35S:FtsH6* construct was generated in the same way. Constructs were transformed by floral dip into *Arabidopsis* using *Agrobacterium tumefaciens*.

### Heat stress treatments

All heat stress treatments were performed in a water bath or incubator, in the dark. Thermomemory experiments were performed as described^65^. For the priming heat stress, seedlings were subjected to a heat regime of 1.5 h, 37 °C; 1.5 h recovery at 22 °C; and 45 min, 44 °C. After the priming heat stress treatment, seedlings were returned to normal growth condition for 3–4 days (recovery or memory phase) and then subjected to heat stress/triggering stimulus, that is, 1.5 h, 44 °C, a treatment normally lethal to plants that have not experienced a priming stimulus. For acquisition of thermotolerance, plants were initially heated to 37 °C for 90 min, returned to normal growth condition for 90 min, before finally heating to 44 °C for 100 min. Basal thermotolerance was assayed by heating plants to 44 °C without prior treatment at 37 °C. After heat treatments, plants were returned to normal growth conditions until analysis.

The quantitative hypocotyl elongation assay was adopted from ref. 42. In our assay, 4-day-old vertically grown, etiolated seedlings were primed at 37 °C for 90 min, then subjected to recovery for 2 days at 22 °C (memory phase), and finally subjected to the triggering heat stress (44 °C, 45 min). The priming heat treatment was omitted in control experiments. Hypocotyl length was measured 5 days after the heat stress.

### Immunoblotting and signal quantification

Total proteins from *Arabidopsis* seedlings were prepared by phenol-based method^66^. Tissue powder was homogenized in 500 μl 0.7 M sucrose/0.5 M Tris/50 mM EDTA/0.1 M potassium chloride, pH 9.4, containing 2% (v/v) 2-mercaptoethanol and cOmplete Protease Inhibitor Cocktail (Roche; diluted to 1 × final concentration) added before use. The homogenate was mixed with 500 μl phenol and centrifuged at 20,000*g* (centrifuge 5427 R, Eppendorf) for 10 min at 4 °C. The upper, phenol phase was taken, and proteins were precipitated with the addition of 1 ml of 0.1 M ammonium acetate in methanol and overnight incubation at −20 °C. The protein pellet obtained following centrifugation was washed sequentially with methanolic ammonium. The protein pellets were dissolved in 1% SDS. The protein concentration was determined with the BCA Protein Assay kit (Thermo Fischer Scientific).

Proteins were resolved by SDS polyacrylamide gel electrophoresis. For immunoblot analysis, proteins were blotted to Protan nitrocellulose membrane (Sigma-Aldrich). Rabbit polyclonal antibodies were used in the following dilutions (v/v): anti-Hsp21 (Abcam; product number: ab80175), 1:3,000; anti-FtsH6 (Agrisera; AS05094A), 1:1,000; anti-HSP101 (Abcam; ab80121), 1:1,000. IRDye 800CW-conjugated goat anti-rabbit IgG(H+L) antibody was used as secondary antibody at 1:10,000 dilution (LI-COR Biosciences; 926-32211). Capturing bands of interest was done using the Odyssey Infrared Imaging System (LI-COR Biosciences), followed by quantifying band density with ImageJ software (www. imagej.net).

In all western blot experiments, loading controls originated from the same gel. For HSP21 and HSP101, the gels were cut into two parts. For HSP21, the lower part was used for immunodetection and the upper part was used for quantification of the protein (RbcL) loading, while for HSP101, the upper part was used for immunodetection and the lower part was used for quantification of RbcL loading. For FtsH6, the membrane was stripped after immunodetection and then stained with Ponceau S (Sigma) for loading control. For the comparisons between *Arabidopsis* Col-0 and N13 accessions, experiments were done in parallel in identical incubation solutions and with identical incubation times and conditions. Gel and immunoblot images have been cropped for presentation. Full-size images are presented in Supplementary Fig. 10.

### Chemical treatments

Five-day-old primed seedlings were transferred to liquid culture medium containing 10 μM cycloheximide, 150 μM 1,10-phenanthroline or 0.1% (v/v) dimethylsulphoxide (mock) as control. The seedlings were harvested at the indicated time points and analysed by immunoblotting.

### RNA isolation and microarray analysis

Global gene expression analysis by Affymetrix was performed in one experiment. The RNeasy Plant Mini kit (Qiagen, Hilden, Germany) was used to extract total RNA from primed and unprimed Col-0 seedlings at different time points (4, 8, 24 and 48 h) into the memory phase. Each sample was a pool of ∼160 seedlings. A 3-μg aliquot of quality-checked total RNA was then processed for use in Affymetrix ATH1 microarray hybridizations. Probe preparation and Affymetrix ATH1 microarray hybridizations were performed by ATLAS Biolabs (Berlin, Germany). Data analysis was performed as reported^67^. GO annotation was done using the Singular Enrichment Analysis tool from AgriGO (http://bioinfo.cau.edu.cn/agriGO)^68^ with the 60 upregulated genes (corrected *P*-value smaller than 0.0015).

### Quantative reverse trancription–PCR

Synthesis of cDNA and quantative reverse trancription–PCR using SYBR Green were done as described^67^,^69^. Data were analysed using the comparative C_t_ method^70^ with *ACTIN2* (*AT3G18780*) as the reference gene. Briefly, the C_t_ value of each gene was normalized to the C_t_ value of the reference gene, revealing delta C_t_ (dC_t_). The level of expression was expressed as the difference between an arbitrary value of 40 and dC_t_, so that a high 40-dC_t_ value indicates high gene expression level. Primers were designed using QuantPrime (www.quantprime.de). PCR reactions were run on an ABI PRISM 7900HT sequence detection system (Applied Biosystems Applera, Darmstadt, Germany). Primers were obtained from Eurofins MWG Operon; sequences are given in Supplementary Table 1.

### Sequence analyses

For sequence analyses, the tools of the National Center for Biotechnology Information (http://www.ncbi.nlm.nih.gov/) and the Arabidopsis Information Resource (http://www.arabidopsis.org/) were used.

### Data availability

Microarray expression data are available from the NCBI Gene Expression Omnibus (GEO) repository (www.ncbi.nlm.nih.gov/geo/) under accession number GSE72949. The authors declare that all other data supporting the findings of this study are available within the article and its supplementary information files or are available from the corresponding author on request.

## Additional Information

**How to cite this article:** Sedaghatmehr, M. *et al*. The plastid metalloprotease FtsH6 and small heat shock protein HSP21 jointly regulate thermomemory in *Arabidopsis*. *Nat. Commun.* 7:12439 doi: 10.1038/ncomms12439 (2016).

## Supplementary Material

Supplementary Information Supplementary Figures 1 - 10 and Supplementary Table 1

Supplementary Data 1 Expression of thermomemory-associated genes (60 up- and 19 down-regulated genes) during the memory phase compared to unprimed controls.

Supplementary Data 2 Expression of nuclear-encoded, plastid localized proteases during the memory phase compared to unprimed controls. FC, fold change.
