# Supplementary material for: The plastid metalloprotease FtsH6 and small heat shock protein HSP21 jointly regulate thermomemory in Arabidopsis
Source: Nat Commun. 2016 Aug 26;7:12439. doi: 10.1038/ncomms12439 (PMC5007455; doi:10.1038/ncomms12439)
Supplement: Supplementary Information — Supplementary Figures 1 - 10 and Supplementary Table 1 [file ncomms12439-s1.pdf]

Supplementary Figure 1

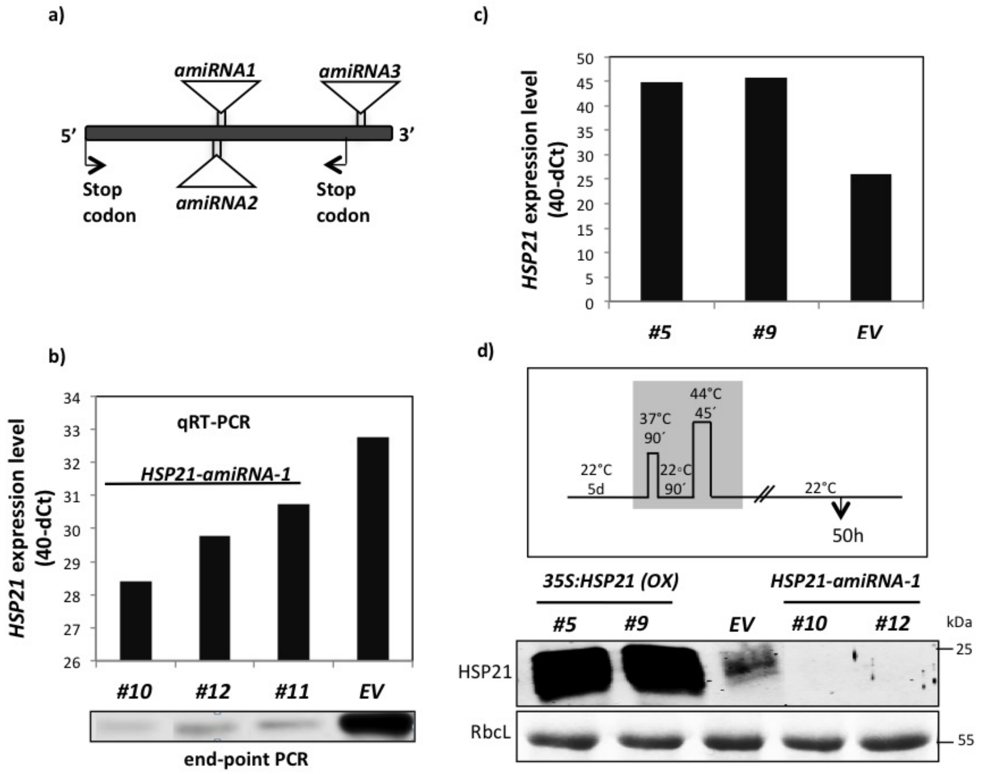

Supplementary Figure 1. *HSP21* expression in *35S:HSP21* and *hsp21* knockdown plants. (a) Since no T-DNA insertion line for *HSP21* is available in the publicly available T-DNA collections, we employed an artificial microRNA (amiRNA)-mediated knockdown strategy to generate plants with reduced *HSP21* gene activity (see Methods). Schematic presentation of the *HSP21* coding sequence showing the positions of different sites targeted by the designed amiRNAs. Of the three amiRNA constructs transformed, only amiRNA1 caused a strong reduction in *HSP21* transcript levels (~4- to 16-fold; determined by qRT-PCR in different lines). **(b)** Reduced *HSP21* transcript abundance in all three lines of *amiRNA-1* seedlings (#10, 11 and 12) confirmed by qRT-PCR (n = 2) and end-point PCR. For end-point PCR, primers annealing to the start and stop regions of *HSP21* were used. *cDNA* was prepared from RNA of leaves of five-day-old heat-treated (37°C for 90 min, then 22°C for 90 min, then 44°C for 45 min) seedlings. **(c)** Elevated expression of *HSP21* in *35S:HSP21* plants (lines #5 and #9) compared to empty-vector (*EV*) control plants. Expression was analysed by qRT-PCR (n = 2). Values were expressed as the difference between an arbitrary value of 40 and dCt, so that high 40 - dCt value indicates high gene expression level. **(d)** Immunodetection of HSP21 protein in *HSP21* transgenics and *EV* plants exposed to the HS regime shown schematically, using anti-HSP21 antibody (top panel). Reduction and increase in HSP21 protein was confirmed in amiRNA and overexpression lines, respectively. RbcL, ribulose-1,5-bis-phosphate carboxylase/ oxygenase large subunit (loading control; bottom panel). kDa, kilo Dalton.

Supplementary Figure 2.

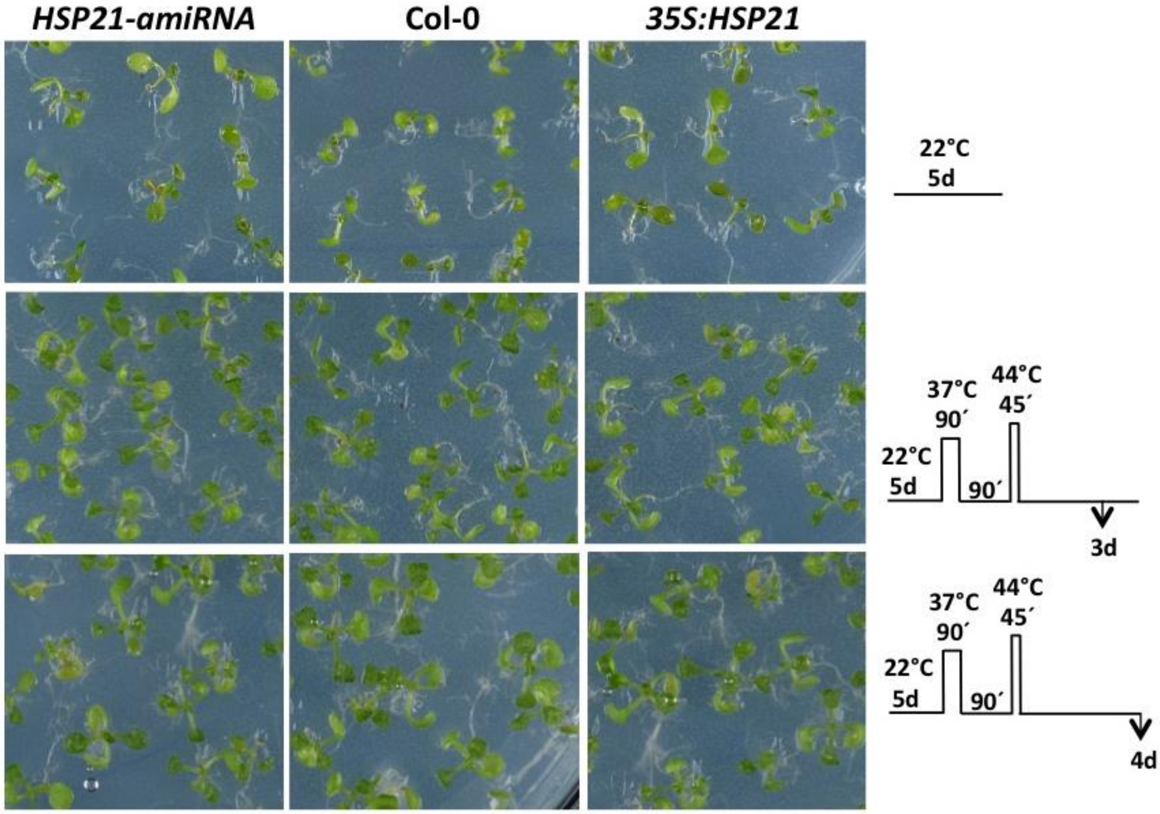

Supplementary Figure 2. Phenotype of *HSP21* transgenic and wild-type (Col-0) plants before and after priming. The phenotype of *HSP21*-amiRNA, *35S:HSP21* and Col-0 plants before heat stress priming (upper row of panels), and after 3 d (middle panels) and 4 d (lower panels) of recovery. Growth condition and heat stress regimes are schematically shown on the right of each row of panels.

Supplementary Figure 3.

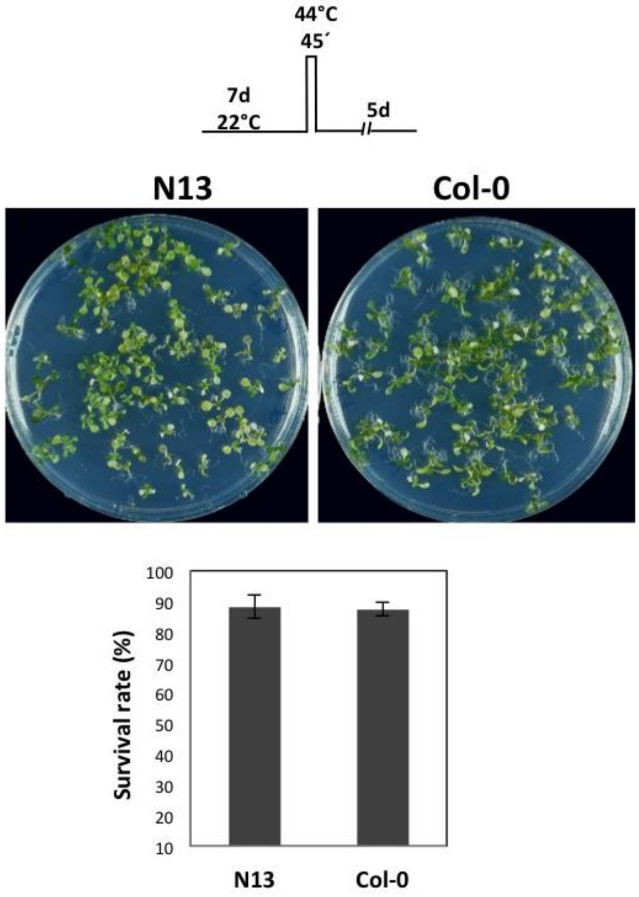

Supplementary Figure 3. Assessment of basal heat tolerance in N13 and Col-0. (a) Seven-day-old N13 and Col-0 seedlings were subjected to 44°C for 45 min. The seedlings were then transferred to normal growth condition and photographed seven days later. **(b)** Seedling survival was scored seven days after the recovery period; data are represented as percentage of the initial viable seedlings. Means ± SD are given (n = 7 plates with ~ 50 seedlings each).

Supplementary Figure 4.

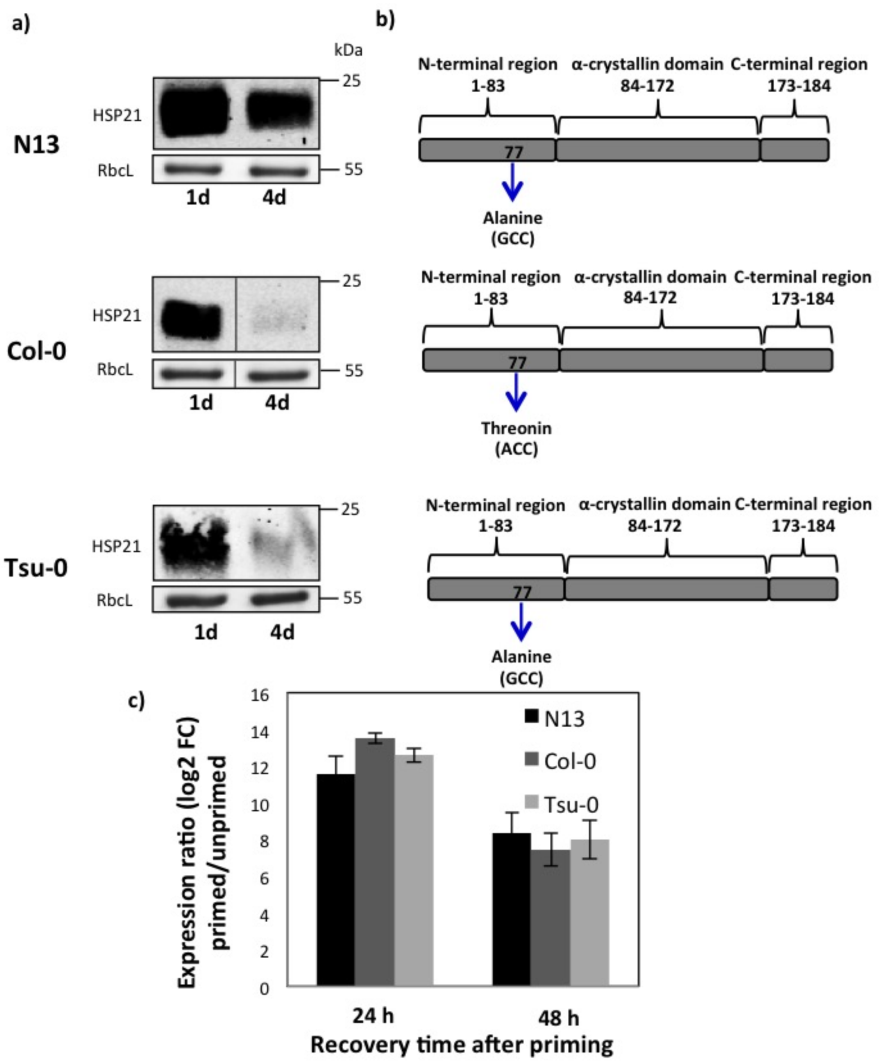

Supplementary Figure 4. Protein level and sequence comparison of HSP21 in *Arabidopsis* accessions N13, Col-0 and Tsu-0. (a) Immunoblot analyses of HSP21 during the memory phase (one and three days after priming) shown on the right of each section. Note the much higher abundance of HSP21 protein level in N13 compared to Col-0 and Tsu-0 at day 4 into the memory phase. kDa, kilo Dalton. Bands shown for Col-0 seedlings have been rearranged for presentation purpose. The original blot image is shown in Supplementary Fig. 10. **(b)** Comparison of the HSP21 protein sequences (deduced from cDNA sequences) in accessions N13, Col-0 and Tsu-0. The threonine 77 in Col-0 is changed to alanine in N13 and Tsu-0 due to an ACC to GCC transition. **(c)** *HSP21* expression in N13, Col-0 and Tsu-0 seedlings during the memory phase (24 h and 48 h) compared to unprimed controls. FC, fold change. Error bars indicate means ± SD of three independent biological replicates each containing a pool of ~100 seedlings.

Supplementary Figure 5. Nucleotide Alignment of *FtsH6* Coding Sequences from *Arabidopsis* Accessions. (a) PCR-amplified *FtsH6* coding sequences of accessions Col-0 and N13 were sequenced and compared. Sequence polymorphisms are shown in yellow. A nucleotide deletion (G) at position 379 of the N13 *FtsH6* open reading frame leads to a premature translation stop codon (red). (b) Alignment of FtsH6 amino acid sequences from *Arabidopsis* accessions.

a) *FtsH6* sequences from accessions Col-0 and N13.

Col-0	1	ATGGCATCATCATCATCAGCTTTGTCTTTCCCTCTCTCCAACATCCCAAC	50
N13	1	ATGGCATCATCATCATCAGCTTTGTCTTTCCCTCTCTCCAACATCCCAAC	50
Col-0	51	CTGTAGTAAGAAGTCTCAACAATTTCAAAAACCGGCTTCTTTATCCAAAT	100
N13	51	CTGTAGTAAGAAGTCTCAACAATTTCAAAAACCGGCTTCTTTATCCAAAT	100
Col-0	101	CCAGTCACACACATAAACCTAGTCTCAAAACCCAAATTTTACACCACAAA	150
N13	101	CCAGTCACACCCATAAACCTAGTCTCAAAACCCAAACTTTACACCACAAA	150
Col-0	151	TTCACTAAGAGAAATTTACTGAGTTTGACGACTGCCTTGGGATTTACGTC	200
N13	151	CTCACTAAGAGAAATTTACTGAGTTTGACGACTGCCTTGGGATTTACGTC	200
Col-0	201	AGCGCTAGGAACTGTTCTTGCTCACCCCGCAAAAGCTGAACCAGAAGCTC	250
N13	201	AGCGCTAGGAACTGTTCTTGCTCACCCCGCAAAAGCTGAACCAGAAGCTC	250
Col-0	251	CCATCGAAGCCACTTCCAATAGAATGTCGTATTCGAGATTCCTGCAGCAT	300
N13	251	CCATCGAAGCCACTTCCAATAGAATGTCGTATTCGAGATTCCTGCAGCAT	300
Col-0	301	CTGAAAGAGAATGAAGTGAAGAAAGTTGACTTGATCGAGAACGGAACGGT	350
N13	301	CTGAAAGAGAATGAAGTGAAGAAAGTTGACTTGATCGAGAACGGAACGGT	350
Col-0	351	TGCGATCGTAGAGATCTCTAATCCAGTAGTAGGAAAGATCCAGAGGGTTA	400
N13	351	TGCGATCGTAGAGATCTCTAATCCAGTA- TAGGAAAGATCCAGAGGGTTA	399
Col-0	401	GGGTTAACCTTCCCGGTTTACCAGTCGATCTGGTGAGGGAGATGAAGGAG	450
N13	400	GGGTTAACCTTCCCGGTTTACCAGTCGATCTGGTGAGGGAGATGAAGGAG	449
Col-0	451	AAGAACGTCGATTTCGCTGCTCATCCCATGAATGTGAACTGGGGAGCTTT	500
N13	450	AAGAACGTCGATTTCGCTGCTCATCCCATGAATGTGAACTGGGGAGCTTT	499
Col-0	501	CTTGCTCAACTTCTTGGGGAATTTAGGGTTTCCTTTGATCTTGCTTGTCT	550
N13	500	CTTGCTCAACTTCTTGGGGAATTTAGGGTTTCCTTTGATCTTGCTTGTCT	549
Col-0	551	CTCTGCTTTTAACATCTTCTTCAAGAAGAAACCCTGCTGGACCTAACTTG	600
N13	550	CTCTGCTTTTAACATCTTCTTCAAGAAGAAACCCTGCTGGACCTAACTTG	599
Col-0	601	CCTTTTGGTCTTGGAAGAAGCAAAGCTAAGTTTCAGATGGAGCCTAACAC	650
N13	600	CCTTTTGGTCTTGGAAGAAGCAAAGCTAAGTTTCAGATGGAGCCTAACAC	649
Col-0	651	AGGGATAACGTTCGAGGATGTAGCAGGAGTAGACGAAGCCAAGCAAGACT	700
N13	650	AGGGATAACGTTCGAGGATGTAGCAGGAGTAGACGAAGCCAAGCAAGACT	699
Col-0	701	TTGAAGAGATCGTTGAATTTTTGAAAACCCCAGAGAAATTCTCAGCTTTG	750
N13	700	TTGAAGAGATCGTTGAATTTTTGAAAACCCCAGAGAAATTCTCAGCTTTG	749
Col-0	751	GGGGCTAAAATCCCAAAAGGCGTCTTGTTGACCGGACCGCCAGGAACCGG	800
N13	750	GGGGCTAAAATCCCAAAAGGCGTCTTGTTGACCGGACCGCCAGGAACCGG	799
Col-0	801	AAAGACACTCTTGGCCAAGGCTATAGCCGGAGAAGCCGGTGTTCCTTTTT	850
N13	800	AAAGACACTCTTGGCCAAGGCTATAGCCGGAGAAGCCGGTGTTCCTTTTT	849
Col-0	851	TTTCATTGTCTGGTTCGGAGTTCATAGAGATGTTTGTTGGTGTAGGAGCA	900

N13	850	TTTCATTGTCTGGTTCGGAGTTCATAGAGATGTTTGTTGGTGTAGGAGCA	899
Col-0	901	TCTAG**A**GCTAGAGACTTGTTTAACAAGGCAAAGGCTAATTCACCTTGTAT	950
N13	900	TCTAG**G**GCTAGAGACTTGTTTAACAAGGCAAAGGCTAATTCACCTTGTAT	949
Col-0	951	AGTGTTCATTGATGAGATTGATGCTGTTGGGAGAATGAGAGGAACCGGTA	1000
N13	950	AGTGTTCATTGATGAGATTGATGCTGTTGGGAGAATGAGAGGAACCGGTA	999
Col-0	1001	TAGGAGGAGGAAACGACGAACGTGAGCAGACGCTAAACCAGATATTAACC	1050
N13	1000	TAGGAGGAGGAAACGACGAACGTGAGCAGACGCTAAACCAGATATTAACC	1049
Col-0	1051	GAAATGGATGGGTTTGCGGGGAATACCGGAGTAATTGTGATCGCTGCAAC	1100
N13	1050	GAAATGGATGGGTTTGCGGGGAATACCGGAGTAATTGTGATCGCTGCAAC	1099
Col-0	1101	GAACCGGCCAGAGATTCTAGACTCTGCTTTGCTCCGACCAGGAAGATTCG	1150
N13	1100	GAACCGGCCAGAGATTCTAGACTCTGCTTTGCTCCGACCAGGAAGATTCG	1149
Col-0	1151	ATAGGCAGGTATGTTGGTTAATCCTTAAACCGAATAAATCGAACAGGTTT	1200
N13	1150	ATAGGCAGGTATGTTGGTTAATCCTTAAACCGAATAAATCGAACAGGTTT	1199
Col-0	1201	GGTATAATGTCAACATGTTTTAAACAGGTTTCTGTTGGTTTACCGGATAT	1250
N13	1200	GGTATAATGTCAACATGTTTTAAACAGGTTTCTGTTGGTTTACCGGATAT	1249
Col-0	1251	AAGAGGAAGAGAGGAGATATTAAAAGTTCACAGCAGAAGCAAGAAACTCG	1300
N13	1250	AAGAGGAAGAGAGGAGATATTAAAAGTTCACAGCAGAAGCAAGAAACTCG	1299
Col-0	1301	ACAAAGATGTATCTT**T**GAGCGTAATTGCCATGAGAACTCCAGGTTTTAGT	1350
N13	1300	ACAAAGATGTATCTT**C**GAGCGTAATTGCCATGAGAACTCCAGGTTTTAGT	1349
Col-0	1351	GGAGCTGACT**T**GGCCAATCTTATGAACGAAGCAGCGATTCTCGCCGGAAG	1400
N13	1350	GGAGCTGACT**A**GGCCAATCTTATGAACGAAGCAGCGATTCTCGCCGGAAG	1399
Col-0	1401	AAGAGGAAAAGACAAGATTACCCTTACAGAGATCGACGACTCTATCGATC	1450
N13	1400	AAGAGGAAAAGACAAGATTACCCTTACAGAGATCGACGACTCTATCGATC	1449
Col-0	1451	GGATAGTCGCCGGAATGGAAGGGACAAAGATGATCGACGGTAAAAGTAAA	1500
N13	1450	GGATAGTCGCCGGAATGGAAGGGACAAAGATGATCGACGGTAAAAGTAAA	1449
Col-0	1501	GCGATTGTAGCGTACCATGAAGTAGGACATGCAATTTGTGCGACTTTGAC	1550
N13	1500	GCGATTGTAGCGTACCATGAAGTAGGACATGCAATTTGTGCGACTTTGAC	1549
Col-0	1551	GGAGGGTCATGATCCGGTTCAGAAAGTTACGTTGGTTCCTAGAGGTCAAG	1600
N13	1550	GGAGGGTCATGATCCGGTTCAGAAAGTTACGTTGGTTCCTAGAGGTCAAG	1559
Col-0	1601	CACGTGGTCTCACGTGGTTCTTACCGGGAGAAGATCCGACATTGGTGTCT	1650
N13	1600	CACGTGGTCTCACGTGGTTCTTACCGGGAGAAGATCCGACATTGGTGTCT	1649
Col-0	1651	AAACAGCAATTGTTCGCTAGAATCGTCGGAGGACTCGGAGGCAGAGCCGC	1700
N13	1650	AAACAGCAATTGTTCGCTAGAATCGTCGGAGGACTCGGAGGCAGAGCCGC	1699
Col-0	1701	CGAAGATGTAATTTTTGGAGAACCGGAGATAACCACCGGCGCTGCCGGTG	1750
N13	1700	CGAAGATGTAATTTTTGGAGAACCGGAGATAACCACCGGCGCTGCCGGTG	1749
Col-0	1751	ATCTCCAGCAAGTCACTGAGATAGCAAGACAGATGGTGACGATGTTTGGT	1800
N13	1750	ATCTCCAGCAAGTCACTGAGATAGCAAGACAGATGGTGACGATGTTTGGT	1799
Col-0	1801	ATGTCGGAAATCGGTCCATGGGCACTAACCGACCCAGCGGTTAAGCAAAA	1850
N13	1800	ATGTCGGAAATCGGTCCATGGGCACTAACCGACCCAGCGGTTAAGCAAAA	1849
Col-0	1851	CGATGTGGTTCTACGGATGCTAGCGAGGAACTCAATGTCCGAGAAACTTG	1900
N13	1850	CGATGTGGTTCTACGGATGCTAGCGAGGAACTCAATGTCCGAGAAACTTG	1899
Col-0	1901	CGGAGGATATTGATTCTTGCGTGAAGAAAATAATCGGTGACGCTTACGAG	1950
N13	1900	CGGAGGATATTGATTCTTGCGTGAAGAAAATAATCGGTGACGCTTACGAG	1949

Col-0<br>N13	1951<br>1950	GTTGCGAAAAAGCACGTGAGGAATAATAGAGAAGCTATAGACAAGCTTGT<br>GTTGCGAAAAAGCACGTGAGGAATAATAGAGAAGCTATAGACAAGCTTGT	2000<br>1999
Col-0<br>N13	2001<br>2000	TGATGTTTTGTTGGAGAAAGAAACCTTAACGGGAGACGAGTTTCGAGCAA<br>TGATGTTTTGTTGGAGAAAGAAACCTTAACGGGAGACGAGTTTCGAGCAA	2050<br>2049
Col-0<br>N13	2051<br>2050	TTCTGTCTGAGTATACTGATCAACCGTTAAATACCGATGGCGATGT**A**AGA<br>TTCTGTCTGAGTATACTGATCAACCGTTAAATACCGATGGCGATGT**T**AGA	2100<br>2099
Col-0<br>N13	2101<br>2100	ATTCGAATCAATGACTTGATTAGTGTCTAA<br>ATTCGAATCAATGACTTGATTAGTGTCTAA	2130<br>2129

FtsH6 protein from Col-0

Met A S S S S A L S F P L S N I P T C S K K S Q Q F Q K P A S L S K S S H T H K P S L K T Q I L H H K F T K R N L L S L T T A L G F T S A L G T V L A H P A K A E P E A P I E A T S N R Met S Y S R F L Q H L K E N E V K K V D L I E N G T V A I V E I S N P V V G K I Q R V R V N L P G L P V D L V R E Met K E K N V D F A A H P Met N V N W G A F L L N F L G N L G F P L I L L V S L L L T S S S R R N P A G P N L P F G L G R S K A K F Q Met E P N T G I T F E D V A G V D E A K Q D F E E I V E F L K T P E K F S A L G A K I P K G V L L T G P P G T G K T L L A K A I A G E A G V P F F S L S G S E F I E Met F V G V G A S R A R D L F N K A K A N S P C I V F I D E I D A V G R Met R G T G I G G G N D E R E Q T L N Q I L T E Met D G F A G N T G V I V I A A T N R P E I L D S A L L R P G R F D R Q V C W L I L K P N K S N R F G I Met S T C F K Q V S V G L P D I R G R E E I L K V H S R S K K L D K D V S L S V I A Met R T P G F S G A D L A N L Met N E A A I L A G R R G K D K I T L T E I D D S I D R I V A G Met E G T K Met I D G K S K A I V A Y H E V G H A I C A T L T E G H D P V Q K V T L V P R G Q A R G L T W F L P G E D P T L V S K Q Q L F A R I V G G L G G R A A E D V I F G E P E I T T G A A G D L Q Q V T E I A R Q Met V T Met F G Met S E I G P W A L T D P A V K Q N D V V L R Met L A R N S Met S E K L A E D I D S C V K K I I G D A Y E V A K K H V R N N R E A I D K L V D V L L E K E T L T G D E F R A I L S E Y T D Q P L N T D G D V R I R I N D L I S V
Stop

FtsH6 protein from N13

Met A S S S S A L S F P L S N I P T C S K K S Q Q F Q K P A S L S K S S H T H K P S L K T Q T L H H K L T K R N L L S L T T A L G F T S A L G T V L A H P A K A E P E A P I E A T S N R Met S Y S R F L Q H L K E N E V K K V D L I E N G T V A I V E I S N P V **Stop**

b) Alignment of FtsH6 amino acid sequences from *Arabidopsis* accessions.

The following *Arabidopsis* accessions were included in our preliminary screen for differences in thermomemory: Col-0, N13, Cvi-0, C24, Ct, Can-0, Lip-0, Mdn-1, Mh-0, Mt-0, Aitba-1, Abd-0, Bl-1, Bur-0, Dog-4, Tsu-0, St-0, Kas-1, Yo-0, Xan-1, Kondara, Ler-0, Ko-2, Ri-0, Fei-0, Leo-1, Yeg-1, Ste-0, ICE1, Kin-0, Le-0, Oy-0, N14, Sah-0, Berk, Hiro, Ita, HEK2, Stepn, Shign, and Borsk2.

The FtsH6 amino acid sequence alignment shown below includes sequences from all above accessions with the exception of sequences that were identical to those of Col-0 (namely Lip-0, Mh-0, Bl-1, Tsu-0,

Ler-0, Ko-2, Fei-0, Ste-0, and Oy-0), or those for which no sequence information was available (N14, Sah-0, Berk, Hiro, Ita, HEK2, Stepn, Shign, and Borsk2). Also not included in the alignment is the sequence of N13, which has a premature stop codon (see above).

FtsH6 sequences were extracted from the Arabidopsis 1001 Genomes project database (http://signal.salk.edu/atg1001/3.0/gebrowser.php). For the multi-sequence alignment the MUSCLE tool (http://www.ebi.ac.uk/Tools/msa/muscle) was used.

Amino acids divergent from Col-0 are highlighted in red. Conservative substitutions are indicated as ´:´ in the Col-0 sequence, while non-conservative exchanges are indicated by a free space. Asterisks (*) denote amino acids identical to Col-0.

```
Can-0           MASSSSALSFPLSNIPTCSKKSQQFQKPASLSKSSHTHKPSLKTQILHHKFTKRNLLSLT
Cvi-0           MASSSSALSFPLSNIPTCSKKSQQFQKPASLSKSSHTHKPSLKTQILHHKFTKRNLLSLT
Bur-0           MASSSSALSFPLSNIPTCSKKSQQFQKPASLSKSSHTHKPSLKTQILHHKFTKRNLLSLT
Leo-1           MASSSSALSFPLSNIPTCSKKSQQFQKPASLSKSSHTHKPSLKTQILHHKFTKRNLLSLT
Le-0            MASSSSALSFPLSNIPTCSKKSQQFQKPASLSKSSHTHKPSLKTQILHHKFTKRNLLSLT
ICE1            MASSSSALSFPLSNIPTCSKKSQQFQKPASLSKSSHTHKPSLKTQILHHKFTKRNLLSLT
Yeg-1           MASSSSALSFPLSNIPTCSKKSQQFQKPASLSKSSHTHKPSLKTQILHHKFTKRNLLSLT
Kondara         MASSSSALSFPLSNIPTCSKKSQQFQKPASLSKSSHTHKPSLKTQILHHKFTKRNLLSLT
Yo-0            MASSSSALSFPLSNIPTCSKKSQQFQKPASLSKSSHTHKPSLKTQILHHKFTKRNLLSLT
Dog-4           MASSSSALSFPLSNIPTCSKKSQQFQKPASLSKSSHTHKPSLKTQILHHKFTKRNLLSLT
Aitba-1         MASSSSALSFPLSNIPTCSKKSQQFQKPASLSKSSHTHKPSLKTQILHHKFTKRNLLSLT
Kin-0           MASSSSALSFPLSNIPTCSKKSQQFQKPASLSKSSHTHKPSLKTQILHHKFTKRNLLSLT
Ri-0            MASSSSALSFPLSNIPTCSKKSQQFQKPASLSKSSHTHKPSLKTQILHHKFTKRNLLSLT
St-0            MASSSSALSFPLSNIPTCSKKSQQFQKPASLSKSSHTHKPSLKTQILHHKFTKRNLLSLT
Abd-0           MASSSSALSFPLSNIPTCSKKSQQFQKPASLSKSSHTHKPSLKTQILHHKFTKRNLLSLT
Mdn-1           MASSSSALSFPLSNIPTCSKKSQQFQKPASLSKSSHTHKPSLKTQILHHKFTKRNLLSLT
Xan-1           MASSSSALSFPLSNIPTCSKKSQQFQNPASLSKSSHTHKPSLKTQILHHKFTKRNLLSLT
Mt-0            MASSSSALSFPLSNIPTCSKKSQQFQNPASLSKSSHTHKPSLKTQILHHKFTKRNLLSLT
Ct              MASSSSALSFPLSNIPTCSKKSQQFQNPASLSKSSHTHKPSLKTQILHHKFTKRNLLSLT
C24             MASSSSALSFPLSNIPTCSKKSQQFQNPASLSKSSHTHKPSLKTQILHHKFTKRNLLSLT
Kas-1           MASSSSALSFPLSNIPTCSKKSQQFQKPASLSKSSHTHKPSLKTQILHHKFTKRNLLSLT
Col-0           MASSSSALSFPLSNIPTCSKKSQQFQKPASLSKSSHTHKPSLKTQILHHKFTKRNLLSLT
                **************************:*********************************

Can-0           TALGFTSALGTVLAHPAKAEPEAPIEATSNRMSYSRFLQHLKENKVKKVDLIENGTVAIV
Cvi-0           TALGFTSALGTVLAHPAKAEPEAPIEATSNRMSYSRFLQHLKENKVKKVDLIENGTVAIV
Bur-0           TALGFTSALGTVLAHPAKAEPEAPIEATSNRMSYSRFLQHLKENEVKKVDLIENGTVAIV
Leo-1           TALGFTSALGTVLAHPAKAEPEAPIEATSNRMSYSRFLQHLKENEVKKVDLIENGTVAIV
Le-0            TALGFTSALGTVLAHPAKAEPEAPIEATSNRMSYSRFLQHLKENEVKKVDLIENGTVAIV
ICE1            TALGFTSALGTVLAHPAKAEPEAPIEATSNRMSYSRFLQHLKENEVKKVDLIENGTVAIV
Yeg-1           TALGFTSALGTVLAHPAKAEPEAPIEATSNRMSYSRFLQHLKENEVKKVDLIENGTVAIV
Kondara         TALGFTSALGTVLAHPAKAEPEAPIEATSNRMSYSRFLQHLKENEVKKVDLIENGTVAIV
Yo-0            TALGFTSALGTVLAHPAKAEPEAPIEATSNRMSYSRFLQHLKENEVKKVDLIENGTVAIV
Dog-4           TALGFTSALGTVLAHPAKAEPEAPIEATSNRMSYSRFLQHLKENEVKKVDLIENGTVAIV
Aitba-1         TALGFTSALGTVLAHPAKAEPEAPIEATSNRMSYSRFLQHLKENEVKKVDLIENGTVAIV
Kin-0           TALGFTSALGTVLAHPAKAEPEAPIEATSNRMSYSRFLQHLKENEVKKVDLIENGTVAIV
Ri-0            TALGFTSALGTVLAHPAKAEPEAPIEATSNRMSYSRFLQHLKENEVKKVDLIENGTVAIV
St-0            TALGFTSALGTVLAHPAKAEPEAPIEATSNRMSYSRFLQHLKENEVKKVDLIENGTVAIV
Abd-0           TALGFTSALGTVLAHPAKAEPEAPIEATSNRMSYSRFLQHLKENEVKKVDLIENGTVAIV
Mdn-1           TALGFTSALGTVLAHPAKAEPEAPIEATSNRMSYSRFLQHLKENEVKKVDLIENGTVAIV
Xan-1           TALGFTSALGTVLAHPAKAEPEAPIEATSNRMSYSRFLQHLKENEVKKVDLIENGTVAIV
Mt-0            TALGFTSALGTVLAHPAKAEPEAPIEATSNRMSYSRFLQHLKENEVKKVDLIENGTVAIV
Ct              TALGFTSALGTVLAHPAKAEPEAPIEATSNRMSYSRFLQHLKENEVKKVDLIENGTVAIV
C24             TALGFTSALGTVLAHPAKAEPEAPIEATSNRMSYSRFLQHLKENEVKKVDLIENGTVAIV
```

```
Kas-1           TALGFTSALGTVLAHPAKAEPEAPIEATSNRMSYSRFLQHLKENEVKKVDLIENGTVAIV
Col-0           TALGFTSALGTVLAHPAKAEPEAPIEATSNRMSYSRFLQHLKENEVKKVDLIENGTVAIV
                ********************************************:***************

Can-0           EISNPVVGKIQRVRVNLPGLPVDLVREMKEKNVDFAAHPMNVNWGAFLLNFLGNLGFPLI
Cvi-0           EISNPVVGKIQRVRVNLPGLPVDLVREMKEKNVDFAAHPMNVNWGAFLLNFLGNLGFPLI
Bur-0           EISNPVVGKIQRVRVNLPGLPVDLVREMKEKNVDFAAHPMNVNWGAFLLNFLGNLGFPLI
Leo-1           EISNPVVGKIQRVRVNLPGLPVDLVREMKEKNVDFAAHPMNVNWGAFLLNFLGNLGFPLI
Le-0            EISNPVVGKIQRVRVNLPGLPVDLVREMKEKNVDFAAHPMNVNWGAFLLNFLGNLGFPLI
ICE1            EISNPVVGKIQRVRVNLPGLPVDLVREMKEKNVDFAAHPMNVNWGAFLLNFLGNLGFPLI
Yeg-1           EISNPVVGKIQRVRVNLPGLPVDLVREMKEKNVDFAAHPMNVNWGAFLLNFLGNLGFPLI
Kondara         EISNPVVGKIQRVRVNLPGLPVDLVREMKEKNVDFAAHPMNVNWGAFLLNFLGNLGFPLI
Yo-0            EISNPVVGKIQRVRVNLPGLPVDLVREMKEKNVDFAAHPMNVNWGAFLLNFLGNLGFPLI
Dog-4           EISNPVVGKIQRVRVNLPGLPVDLVREMKEKNVDFAAHPMNVNWGAFLLNFLGNLGFPLI
Aitba-1         EISNPVVGKIQRVRVNLPGLPVDLVREMKEKNVDFAAHPMNVNWGAFLLNFLGNLGFPLI
Kin-0           EISNPVVGKIQRVRVNLPGLPVDLVREMKEKNVDFAAHPMNVNWGAFLLNFLGNLGFPLI
Ri-0            EISNPVVGKIQRVRVNLPGLPVDLVREMKEKNVDFAAHPMNVNWGAFLLNFLGNLGFPLI
St-0            EISNPVVGKIQRVRVNLPGLPVDLVREMKEKNVDFAAHPMNVNWGAFLLNFLGNLGFPLI
Abd-0           EISNPVVGKIQRVRVNLPGLPVDLVREMKEKNVDFAAHPMNVNWGAFLLNFLGNLGFPLI
Mdn-1           EISNPVVGKIQRVRVNLPGLPVDLVREMKEKNVDFAAHPMNVNWGAFLLNFLGNLGFPLI
Xan-1           EISNPVVGKIQRVRVNLPGLPVDLVREMKEKNVDFAAHPMNVNWGAFLLNFLGNLGFPLI
Mt-0            EISNPVVGKIQRVRVNLPGLPVDLVREMKEKNVDFAAHPMNVNWGAFLLNFLGNLGFPLI
Ct              EISNPVVGKIQRVRVNLPGLPVDLVREMKEKNVDFAAHPMNVNWGAFLLNFLGNLGFPLI
C24             EISNPVVGKIQRVRVNLPGLPVDLVREMKEKNVDFAAHPMNVNWGAFLLNFLGNLGFPLI
Kas-1           EISNPVVGKIQRVRVNLPGLPVDLVREMKEKNVDFAAHPMNVNWGAFLLNFLGNLGFPLI
Col-0           EISNPVVGKIQRVRVNLPGLPVDLVREMKEKNVDFAAHPMNVNWGAFLLNFLGNLGFPLI
                ************************************************************

Can-0           LLVSLLLTSSSRRNPAEPNLPFGLGRSKAKFQMEPNTGITFEDVAGVDEAKQDFEEIVEF
Cvi-0           LLVSLLLTSSSRRNPAEPNLPFGLGRSKAKFQMEPNTGITFEDVAGVDEAKQDFEEIVEF
Bur-0           LLVSLLLTSSSRRNPAGPNLPFGLGRSKAKFQMEPNTGITFEDVAGVDEAKQDFEEIVEF
Leo-1           LLVSLLLTSSSRRNPAGPNLPFGLGRSKAKFQMEPNTGITFEDLAGVDEAKQDFEEIVEF
Le-0            LLVSLLLTSSSRRNPAGPNLPFGLGRSKAKFQMEPNTGITFEDVAGVDEAKQDFEEIVEF
ICE1            LLVSLLLTSSSRRNPAGPNLPFGLGRSKAKFQMEPNTGITFEDVAGVDEAKQDFEEIVEF
Yeg-1           LLVSLLLTSSSRRNPAGPNLPFGLGRSKAKFQMEPNTGITFEDVAGVDEAKQDFEEIVEF
Kondara         LLVSLLLTSSSRRNPAGPNLPFGLGRSKAKFQMEPNTGITFEDVAGVDEAKQDFEEIVEF
Yo-0            LLVSLLLTSSSRRNPAGPNLPFGLGRSKAKFQMEPNTGITFEDVAGVDEAKQDFEEIVEF
Dog-4           LLVSLLLTSSSRRNPAGPNLPFGLGRSKAKFQMEPNTGITFEDVAGVDEAKQDFEEIVEF
Aitba-1         LLVSLLLTSSSRRNPAGPNLPFGLGRSKAKFQMEPNTGITFEDVAGVDEAKQDFEEIVEF
Kin-0           LLVSLLLTSSSRRNPAGPNLPFGLGRSKAKFQMEPNTGITFEDVAGVDEAKQDFEEIVEF
Ri-0            LLVSLLLTSSSRRNPAGPNLPFGLGRSKAKFQMEPNTGITFEDVAGVDEAKQDFEEIVEF
St-0            LLVSLLLTSSSRRNPAGPNLPFGLGRSKAKFQMEPNTGITFEDVAGVDEAKQDFEEIVEF
Abd-0           LLVSLLLTSSSRRNPAGPNLPFGLGRSKAKFQMEPNTGITFEDVAGVDEAKQDFEEIVEF
Mdn-1           LLVSLLLTSSSRRNPAGPNLPFGLGRSKAKFQMEPNTGITFEDVAGVDEAKQDFEEIVEF
Xan-1           LLVSLLLTSSSRRNPAGPNLPFGLGRSKAKFQMEPNTGITFEDVAGVDEAKQDFEEIVEF
Mt-0            LLVSLLLTSSSRRNPAGPNLPFGLGRSKAKFQMEPNTGITFEDVAGVDEAKQDFEEIVEF
Ct              LLVSLLLTSSSRRNPAGPNLPFGLGRSKAKFQMEPNTGITFEDVAGVDEAKQDFEEIVEF
C24             LLVSLLLTSSSRRNPAGPNLPFGLGRSKAKFQMEPNTGITFEDVAGVDEAKQDFEEIVEF
Kas-1           LLVSLLLTSSSRRNPAGPNLPFGLGRSKAKFQMEPNTGITFEDVAGVDEAKQDFEEIVEF
Col-0           LLVSLLLTSSSRRNPAGPNLPFGLGRSKAKFQMEPNTGITFEDVAGVDEAKQDFEEIVEF
                **************** **************************:****************

Can-0           LKTPEKFSALGAKIPKGVLLTGPPGTGKTLLAKAIAGEAGVPLFSLSGSEFIEMFVGVGA
Cvi-0           LKTPEKFSALGAKIPKGVLLTGPPGTGKTLLAKAIAGEAGVPLFSLSGSEFIEMFVGVGA
Bur-0           LKTPEKFSALGAKIPKGVLLTGPPGTGKTLLAKAIAGEAGVPFFSLSGSEFIEMFVGVGA
Leo-1           LKTPEKFSALGAKIPKGVLLTGPPGTGKTLLAKAIAGEAGVPFFSLSGSEFIEMFVGVGA
Le-0            LKTPEKFSALGAKIPKGVLLTGPPGTGKTLLAKAIAGEAGVPFFSLSGSEFIEMFVGVGA
ICE1            LKTPEKFSALGAKIPKGVLLTGPPGTGKTLLAKAIAGEAGVPFFSLSGSEFIEMFVGVGA
Yeg-1           LKTPEKFSALGAKIPKGVLLTGPPGTGKTLLAKAIAGEAGVPFFSLSGSEFIEMFVGVGA
Kondara         LKTPEKFSALGAKIPKGVLLTGPPGTGKTLLAKAIAGEAGVPFFSLSGSEFIEMFVGVGA
Yo-0            LKTPEKFSALGAKIPKGVLLTGPPGTGKTLLAKAIAGEAGVPFFSLSGSEFIEMFVGVGA
Dog-4           LKTPEKFSALGAKIPKGVLLTGPPGTGKTLLAKAIAGEAGVPFFSLSGSEFIEMFVGVGA
Aitba-1         LKTPEKFSALGAKIPKGVLLTGPPGTGKTLLAKAIAGEAGVPFFSLSGSEFIEMFVGVGA
```

```
Kin-0           LKTPEKFSALGAKIPKGVLLTGPPGTGKTLLAKAIAGEAGVPFFSLSGSEFIEMFVGVGA
Ri-0            LKTPEKFSALGAKIPKGVLLTGPPGTGKTLLAKAIAGEAGVPFFSLSGSEFIEMFVGVGA
St-0            LKTPEKFSALGAKIPKGVLLTGPPGTGKTLLAKAIAGEAGVPFFSLSGSEFIEMFVGVGA
Abd-0           LKTPEKFSALGAKIPKGVLLTGPPGTGKTLLAKAIAGEAGVPFFSLSGSEFIEMFVGVGA
Mdn-1           LKTPEKFSALGAKIPKGVLLTGPPGTGKTLLAKAIAGEAGVPFFSLSGSEFIEMFVGVGA
Xan-1           LKTPEKFSALGAKIPKGVLLTGPPGTGKTLLAKAIAGEAGVPFFSLSGSEFIEMFVGVGA
Mt-0            LKTPEKFSALGAKIPKGVLLTGPPGTGKTLLAKAIAGEAGVPFFSLSGSEFIEMFVGVGA
Ct              LKTPEKFSALGAKIPKGVLLTGPPGTGKTLLAKAIAGEAGVPFFSLSGSEFIEMFVGVGA
C24             LKTPEKFSALGAKIPKGVLLTGPPGTGKTLLAKAIAGEAGVPFFSLSGSEFIEMFVGVGA
Kas-1           LKTPEKFSALGAKIPKGVLLTGPPGTGKTLLAKAIAGEAGVPFFSLSGSEFIEMFVGVGA
Col-0           LKTPEKFSALGAKIPKGVLLTGPPGTGKTLLAKAIAGEAGVPFFSLSGSEFIEMFVGVGA
                ******************************************:*****************

Can-0           SRARDLFNKAKANSPCIVFIDEIDAVGRMRGTGIGGGNDEREQTLNQILTEMDGFAGNTG
Cvi-0           SRARDLFNKAKANSPCIVFIDEIDAVGRMRGTGIGGGNDEREQTLNQILTEMDGFAGNTG
Bur-0           SRARDLFNKAKANSPCIVFIDEIDAVGRMRGTGIGGGNDEREQTLNQILTEMDGFAGNTG
Leo-1           SRARDLFNKAKANSPCIVFIDEIDAVGRMRGTGIGGGNDEREQTLNQILTEMDGFAGNTG
Le-0            SRARDLFNKAKANSPCIVFIDEIDAVGRMRGTGIGGGNDEREQTLNQILTEMDGFAGNTG
ICE1            SRARDLFNKAKANSPCIVFIDEIDAVGRMRGTGIGGGNDEREQTLNQILTEMDGFAGNTG
Yeg-1           SRARDLFNKAKANSPCIVFIDEIDAVGRMRGTGIGGGNDEREQTLNQILTEMDGFAGNTG
Kondara         SRARDLFNKAKANSPCIVFIDEIDAVGRMRGTGIGGGNDEREQTLNQILTEMDGFAGNTG
Yo-0            SRARDLFNKAKANSPCIVFIDEIDAVGRMRGTGIGGGNDEREQTLNQILTEMDGFAGNTG
Dog-4           SRARDLFNKAKANSPCIVFIDEIDAVGRMRGTGIGGGNDEREQTLNQILTEMDGFAGNTG
Aitba-1         SRARDLFNKAKANSPCIVFIDEIDAVGRMRGTGIGGGNDEREQTLNQILTEMDGFAGNTG
Kin-0           SRARDLFNKAKANSPCIVFIDEIDAVGRMRGTGIGGGNDEREQTLNQILTEMDGFAGNTG
Ri-0            SRARDLFNKAKANSPCIVFIDEIDAVGRMRGTGIGGGNDEREQTLNQILTEMDGFAGNTG
St-0            SRARDLFNKAKANSPCIVFIDEIDAVGRMRGTGIGGGNDEREQTLNQILTEMDGFAGNTG
Abd-0           SRARDLFNKAKANSPCIVFIDEIDAVGRMRGTGIGGGNDEREQTLNQILTEMDGFAGNTG
Mdn-1           SRARDLFNKAKANSPCIVFIDEIDAVGRMRGTGIGGGNDEREQTLNQILTEMDGFAGNTG
Xan-1           SRARDLFNKAKANSPCIVFIDEIDAVGRMRGTGIGGGNDEREQTLNQILTEMDGFAGNTG
Mt-0            SRARDLFNKAKANSPCIVFIDEIDAVGRMRGTGIGGGNDEREQTLNQILTEMDGFAGNTG
Ct              SRARDLFNKAKANSPCIVFIDEIDAVGRMRGTGIGGGNDEREQTLNQILTEMDGFAGNTG
C24             SRARDLFNKAKANSPCIVFIDEIDAVGRMRGTGIGGGNDEREQTLNQILTEMDGFAGNTG
Kas-1           SRARDLFNKAKANSPCIVFIDEIDAVGRMRGTGIGGGNDEREQTLNQILTEMDGFAGNTG
Col-0           SRARDLFNKAKANSPCIVFIDEIDAVGRMRGTGIGGGNDEREQTLNQILTEMDGFAGNTG
                ************************************************************

Can-0           VIVIAATNRPEILDSALLRPGRFDRQVCWLILKPNKSNRFGIMSTCFKQVSVGLPDIRGR
Cvi-0           VIVIAATNRPEILDSALLRPGRFDRQVCWLILKPNKSNRFGIMSTCFKQVSVGLPDIRGR
Bur-0           VIVIAATNRPEILDSALLRPGRFDRQVCWLILKPNKSNRFGIMSTCFKQVSVGLPDIRGR
Leo-1           VIVIAATNRPEILDSALLRPGRFDRQVCWLILKPNKSNRFGIMSTCFKQVSVGLPDIRGR
Le-0            VIVIAATNRPEILDSALLRPGRFDRQVCWLILKPNKSNRLGIMSTCFKQVSVGLPDIRGR
ICE1            VIVIAATNRPEILDSALLRPGRFDRQVCWLILKPNKSNRLGIMSTCFKQVSVGLPDIRGR
Yeg-1           VIVIAATNRPEILDSALLRPGRFDRQVCWLILKPNKSNRLGIMSTCFKQVSVGLPDIRGR
Kondara         VIVIAATNRPEILDSALLRPGRFDRQVCWLILKPNKSNRLGIMSTCFKQVSVGLPDIRGR
Yo-0            VIVIAATNRPEILDSALLRPGRFDRQVCWLILKPNKSNRLGIMSTCFKQVSVGLPDIRGR
Dog-4           VIVIAATNRPEILDSALLRPGRFDRQVCWLILKPNKSNRLGIMSTCFKQVSVGLPDIRGR
Aitba-1         VIVIAATNRPEILDSALLRPGRFDRQVCWLILKPNKSNRFGIMSTCFKQVSVGLPDIRGR
Kin-0           VIVIAATNRPEILDSALLRPGRFDRQVCWLILKPNKSNRFCIMSTCFKQVSVGLPDIRGR
Ri-0            VIVIAATNRPEILDSALLRPGRFDRQVCWLILKPNKSNRFCIMSTCFKQVSVGLPDIRGR
St-0            VIVIAATNRPEILDSALLRPGRFDRQVCWLILKPNKSNRFCIMSTCFKQVSVGLPDIRGR
Abd-0           VIVIAATNRPEILDSALLRPGRFDRQVCWLILKPNKSNRFCIMSTCFKQVSVGLPDIRGR
Mdn-1           VIVIAATNRPEILDSALLRPGRFDRQVCWLILKPNKSNRFCIMSTCFKQVSVGLPDIRGR
Xan-1           VIVIAATNRPEILDSALLRPGRFDRQVCWLILKPNKSNRFGIMSTCFKQVSVGLPDIRGR
Mt-0            VIVIAATNRPEILDSALLRPGRFDRQVCWLILKPNKSNRFGIMSTCFKQVSVGLPDIRGR
Ct              VIVIAATNRPEILDSALLRPGRFDRQVCWLILKPNKSNRFGIMSTCFKQVSVGLPDIRGR
C24             VIVIAATNRPEILDSALLRPGRFDRQVCWLILKPNKSNRFGIMSTCFKQVSVGLPDIRGR
Kas-1           VIVIAATNRPEILDSALLRPGRFDRQVCWLILKPNKSNRFGIMSTCFKQVSVGLPDIRGR
Col-0           VIVIAATNRPEILDSALLRPGRFDRQVCWLILKPNKSNRFGIMSTCFKQVSVGLPDIRGR
                ***************************************: *******************

Can-0           EEILKVHSRSKKLDKDVSLSVIAMRTPGFSGADLANLMNEAAILAGRRGKDKITLTEIDD
Cvi-0           EEILKVHSRSKKLDKDVSLSVIAMRTPGFSGADLANLMNEAAILAGRRGKDKITLTEIDD
```

```
Bur-0         EEILKVHSRSKKLDKDVSLSVIAMRTPGFSGADLANLMNEAAILAGRRGKDKITLTEIDD
Leo-1         EEILKVHSRSKKLDKDVSSSVIAMRTPGFSGADLANLMNEAAILAGRRGKDKITLTEIDD
Le-0          EEILKVHSRSKKLDKDVSSSVIAMRTPGFSGADLANLMNEAAILAGRRGKDKITLTEIDD
ICE1          EEILKVHSRSKKLDKDVSSSVIAMRTPGFSGADLANLMNEAAILAGRRGKDKITLTEIDD
Yeg-1         EEILKVHSRSKKLDKDVSSSVIAMRTPGFSGADLANLMNEAAILAGRRGKDKITLTEIDD
Kondara       EEILKVHSRSKKLDKDVSSSVIAMRTPGFSGADLANLMNEAAILAGRRGKDKITLTEIDD
Yo-0          EEILKVHSRSKKLDKDVSSSVIAMRTPGFSGADLANLMNEAAILAGRRGKDKITLTEIDD
Dog-4         EEILKVHSRSKKLDKDVSSSVIAMRTPGFSGADLANLMNEAAILAGRRGKDKITLTEIDD
Aitba-1       EEILKVHSRSKKLDKDVSSSVIAMRTPGFSGADLANLMNEAAILAGRRGKDKITLTEIDD
Kin-0         EEILKVHSRSKKLDKDVSLSVIAMRTPGFSGADLANLMNEAAILAGRRGKDKITLTEIDD
Ri-0          EEILKVHSRSKKLDKDVSLSVIAMRTPGFSGADLANLMNEAAILAGRRGKDKITLTEIDD
St-0          EEILKVHSRSKKLDKDVSLSVIAMRTPGFSGADLANLMNEAAILAGRRGKDKITLTEIDD
Abd-0         EEILKVHSRSKKLDKDVSLSVIAMRTPGFSGADLANLMNEAAILAGRRGKDKITLTEIDD
Mdn-1         EEILKVHSRSKKLDKDVSLSVIAMRTPGFSGADLANLMNEAAILAGRRGKDKITLTEIDD
Xan-1         EEILKVHSRSKKLDKDVSLSVIAMRTPGFSGADLANLMNEAAILAGRRGKDKITLTEIDD
Mt-0          EEILKVHSRSKKLDKDVSLSVIAMRTPGFSGADLANLMNEAAILAGRRGKDKITLTEIDD
Ct            EEILKVHSRSKKLDKDVSLSVIAMRTPGFSGADLANLMNEAAILAGRRGKDKITLTEIDD
C24           EEILKVHSRSKKLDKDVSLSVIAMRTPGFSGADLANLMNEAAILAGRRGKDKITLTEIDD
Kas-1         EEILKVHSRSKKLDKDVSLSVIAMRTPGFSGADLANLMNEAAILAGRRGKDKITLTEIDD
Col-0         EEILKVHSRSKKLDKDVSLSVIAMRTPGFSGADLANLMNEAAILAGRRGKDKITLTEIDD
              ****************** *****************************************

Can-0         SIDRIVAGMEGTKMIDGKSKAIVAYHEVGHAICATLTEGHDPVQKVTLVPRGQARGLTWF
Cvi-0         SIDRIVAGMEGTKMIDGKSKAIVAYHEVGHAICATLTEGHDPVQKVTLVPRGQARGLTWF
Bur-0         SIDRIVAGMEGTKMIDGKSKAIVAYHEVGHAICATLTEGHDPVQKVTLVPRGQARGLTWF
Leo-1         SIDRIVAGMEGTKMIDGKSKAIVAYHEVGHAICATLTEGHDPDQKVTLVPRGQARGLTWF
Le-0          SIDRIVAGMEGTKMIDGKSKAIVAYHEVGHAICATLTEGHDPVQKVTLVPRGQARGLTWF
ICE1          SIDRIVAGMEGTKMIDGKSKAIVAYHEVGHAICATLTEGHDPVQKVTLVPRGQARGLTWF
Yeg-1         SIDRIVAGMEGTKMIDGKSKAIVAYHEVGHAICATLTEGHDPVQKVTLVPRGQARGLTWF
Kondara       SIDRIVAGMEGTKMIDGKSKAIVAYHEVGHAICATLTEGHDPVQKVTLVPRGQARGLTWF
Yo-0          SIDRIVAGMEGTKMIDGKSKAIVAYHEVGHAICATLTEGHDPVQKVTLVPRGQARGLTWF
Dog-4         SIDRIVAGMEGTKMIDGKSKAIVAYHEVGHAICATLTEGHDPVQKVTLVPRGQARGLTWF
Aitba-1       SIDRIVAGMEGTKMIDGKSKAIVAYHEVGHAICATLTEGHDPVQKVTLVPRGQARGLTWF
Kin-0         SIDRIVAGMEGTKMIDGKSKAIVAYHEVGHAICATLTEGHDPVQKVTLVPRGQARGLTWF
Ri-0          SIDRIVAGMEGTKMIDGKSKAIVAYHEVGHAICATLTEGHDPVQKVTLVPRGQARGLTWF
St-0          SIDRIVAGMEGTKMIDGKSKAIVAYHEVGHAICATLTEGHDPVQKVTLVPRGQARGLTWF
Abd-0         SIDRIVAGMEGTKMIDGKSKAIVAYHEVGHAICATLTEGHDPVQKVTLVPRGQARGLTWF
Mdn-1         SIDRIVAGMEGTKMIDGKSKAIVAYHEVGHAICATLTEGHDPVQKVTLVPRGQARGLTWF
Xan-1         SIDRIVAGMEGTKMIDGKSKAIVAYHEVGHAICATLTEGHDPVQKVTLVPRGQARGLTWF
Mt-0          SIDRIVAGMEGTKMIDGKSKAIVAYHEVGHAICATLTEGHDPVQKVTLVPRGQARGLTWF
Ct            SIDRIVAGMEGTKMIDGKSKAIVAYHEVGHAICATLTEGHDPVQKVTLVPRGQARGLTWF
C24           SIDRIVAGMEGTKMIDGKSKAIVAYHEVGHAICATLTEGHDPVQKVTLVPRGQARGLTWF
Kas-1         SIDRIVAGMEGTKMIDGKSKAIVAYHEVGHAICATLTEGYDPVQKVTLVPRGQARGLTWF
Col-0         SIDRIVAGMEGTKMIDGKSKAIVAYHEVGHAICATLTEGHDPVQKVTLVPRGQARGLTWF
              ***************************************:** *****************

Can-0         LPGEDPTLVSKQQLFARIVGGLGGRAAEDVIFGEPEITTGAAGDLQQVTEIARQMVTMFG
Cvi-0         LPGEDPTLVSKQQLFARIVGGLGGRAAEDVIFGEPEITTGAAGDLQQVTEIARQMVTMFG
Bur-0         LPGEDPTLVSKQQLFARIVGGLGGRAAEDVIFGEPEITTGAAGDLQQVTEIARQMVTMFG
Leo-1         LPGEDPTLVSKQQLFARIVGGLGGRAAEDVIFGEPEITTGAAGDLQQVTEIARQMVTMFG
Le-0          LPGEDPTLVSKQQLFARIVGGLGGRAAEDVIFGEPEITTGAAGDLQQVTEIARQMVTMFG
ICE1          LPGEDPTLVSKQQLFARIVGGLGGRAAEDVIFGEPEITTGAAGDLQQVTEIARQMVTMFG
Yeg-1         LPGEDPTLVSKQQLFARIVGGLGGRAAEDVIFGEPEITTGAAGDLQQVTEIARQMVTMFG
Kondara       LPGEDPTLVSKQQLFARIVGGLGGRAAEDVIFGEPEITTGAAGDLQQVTEIARQMVTMFG
Yo-0          LPGEDPTLVSKQQLFARIVGGLGGRAAEDVIFGEPEITTGAAGDLQQVTEIARQMVTMFG
Dog-4         LPGEDPTLVSKQQLFARIVGGLGGRAAEDVIFGEPEITTGAAGDLQQVTEIARQMVTMFG
Aitba-1       LPGEDPTLVSKQQLFARIVGGLGGRAAEDVIFGEPEITTGAAGDLQQVTEIARQMVTMFG
Kin-0         LPGEDPTLVSKQQLFARIVGGLGGRAAEDVIFGEPEITTGAAGDLQQVTEIARQMVTMFG
Ri-0          LPGEDPTLVSKQQLFARIVGGLGGRAAEDVIFGEPEITTGAAGDLQQVTEIARQMVTMFG
St-0          LPGEDPTLVSKQQLFARIVGGLGGRAAEDVIFGEPEITTGAAGDLQQVTEIARQMVTMFG
Abd-0         LPGEDPTLVSKQQLFARIVGGLGGRAAEDVIFGEPEITTGAAGDLQQVTEIARQMVTMFG
Mdn-1         LPGEDPTLVSKQQLFARIVGGLGGRAAEDVIFGEPEITTGAAGDLQQVTEIARQMVTMFG
Xan-1         LPGEDPTLVSKQQLFARIVGGLGGRAAEDVIFGEPEITTGAAGDLQQVTEIARQMVTMFG
```

```
Mt-0        LPGEDPTLVSKQQLFARIVGGLGGRAAEDVIFGEPEITTGAAGDLQQVTEIARQMVTMFG
Ct          LPGEDPTLVSKQQLFARIVGGLGGRAAEDVIFGEPEITTGAAGDLQQVTEIARQMVTMFG
C24         LPGEDPTLVSKQQLFARIVGGLGGRAAEDVIFGEPEITTGAAGDLQQVTEIARQMVTMFG
Kas-1       LPGEDPTLVSKQQLFARIVGGLGGRAAEDVIFGEPEITTGAAGDLQQVTEIARQMVTMFG
Col-0       LPGEDPTLVSKQQLFARIVGGLGGRAAEDVIFGEPEITTGAAGDLQQVTEIARQMVTMFG
            ************************************************************

Can-0       MSEIGPWALTDPAVKQNDVVLRMLARNSMSEKLAEDIDSCVKKIIGDAYEVAKKHVRNNR
Cvi-0       MSEIGPWALTDPAVKQNDVVLRMLARNSMSEKLAEDIDSCVKKIIGDAYEVAKKHVRNNR
Bur-0       MSEIGPWALIDPAVKQNDVVLRMLARNSVSEKLAEDIDSCVKKIIGDAYEVAKKHVRNNR
Leo-1       MSEIGPWALTDPAVKQNDVVLRMLARNSMSEKLAEDIDSCVKKIIGDAYEVAKKHVRNNR
Le-0        MSEIGPWALTDPAVKQNDVVLRMLARNSMSEKLAEDIDSCVKKIIGDAYEVAKKHVRNNR
ICE1        MSEIGPWALTDPAVKQNDVVLRMLARNSMSEKLAEDIDSCVKKIIGDAYEVAKKHVRNNR
Yeg-1       MSEIGPWALTDPAVKQNDVVLRMLARNSMSEKLAEDIDSCVKKIIGDAYEVAKKHVRNNR
Kondara     MSEIGPWALTDPAVKQNDVVLRMLARNSMSEKLAEDIDSCVKKIIGDAYEVAKKHVRNNR
Yo-0        MSEIGPWALTDPAVKQNDVVLRMLARNSMSEKLAEDIDSCVKKIIGDAYEVAKKHVRNNR
Dog-4       MSEIGPWALTDPAVKQNDVVLRMLARNSMSEKLAEDIDSCVKKIIGDAYEVAKKHVRNNR
Aitba-1     MSEIGPWALTDPAVKQNDVVLRMLARNSMSEKLAEDIDSCVKKIIGDAYEVAKKHVRNNR
Kin-0       MSEIGPWALTDPAVKQNDVVLRMLARNSMSEKLAEDIDSCVKKIIGDAYEVAKKHVRNNR
Ri-0        MSEIGPWALTDPAVKQNDVVLRMLARNSMSEKLAEDIDSCVKKIIGDAYEVAKKHVRNNR
St-0        MSEIGPWALTDPAVKQNDVVLRMLARNSMSEKLAEDIDSCVKKIIGDAYEVAKKHVRNNR
Abd-0       MSEIGPWALTDPAVKQNDVVLRMLARNSMSEKLAEDIDSCVKKIIGDAYEVAKKHVRNNR
Mdn-1       MSEIGPWALTDPAVKQNDVVLRMLARNSMSEKLAEDIDSCVKKIIGDAYEVAKKHVRNNR
Xan-1       MSEIGPWALTDPAVKQNDVVLRMLARNSMSEKLAEDIDSCVKKIIGDAYEVAKKHVRNNR
Mt-0        MSEIGPWALTDPAVKQNDVVLRMLARNSMSEKLAEDIDSCVKKIIGDAYEVAKKHVRNNR
Ct          MSEIGPWALTDPAVKQNDVVLRMLARNSMSEKLAEDIDSCVKKIIGDAYEVAKKHVRNNR
C24         MSEIGPWALTDPAVKQNDVVLRMLARNSMSEKLAEDIDSCVKKIIGDAYEVAKKHVRNNR
Kas-1       MSEIGPWALTDPAVKQNDVVLRMLARNSMSEKLAEDIDSCVKKIIGDAYEVAKKHVRNNR
Col-0       MSEIGPWALTDPAVKQNDVVLRMLARNSMSEKLAEDIDSCVKKIIGDAYEVAKKHVRNNR
            ********* ******************:*******************************

Can-0       EAIDKLVDVLLEKETLTGDEFRAILSEYTDQPLNTDGDVRIRINDLISV
Cvi-0       EAIDKLVDVLLEKETLTGDEFRAILSEYTDQPLNTDGDVRIRINDLISV
Bur-0       EAIDKLVDVLLEKETLTGDEFRAILSEYTDQPLNTDGDVRIRINDLISV
Leo-1       EAIDKLVDVLLEKETLTGDEFRAILSEYTDQPLNTDGDVRIRINDLISV
Le-0        EAIDKLVDVLLEKETLTGDEFRAILSEYTDQPLNTDGDVRIRINDLISV
ICE1        EAIDKLVDVLLEKETLTGDEFRAILSEYTDQPLNTDGDVRIRINDLISV
Yeg-1       EAIDKLVDVLLEKETLTGDEFRAILSEYTDQPLNTDGDVRIRINDLISV
Kondara     EAIDKLVDVLLEKETLTGDEFRAILSEYTDQPLNTDGDVRIRINDLISV
Yo-0        EAIDKLVDVLLEKETLTGDEFRAILSEYTDQPLNTDGDVRIRINDLISV
Dog-4       EAIDKLVDVLLEKETLTGDEFRAILSEYTDQPLNTDGDVRIRINDLISV
Aitba-1     EAIDKLVDVLLEKETLTGDEFRAILSEYTDQPLNTDGDVRIRINDLISV
Kin-0       EAIDKLVDVLLEKETLTGDEFRAILSEYTDQPLNTDGDVRIRINDLISV
Ri-0        EAIDKLVDVLLEKETLTGDEFRAILSEYTDQPLNTDGDVRIRINDLISV
St-0        EAIDKLVDVLLEKETLTGDEFRAILSEYTDQPLNTDGDVRIRINDLISV
Abd-0       EAIDKLVDVLLEKETLTGDEFRAILSEYTDQPLNTDGDVRIRINDLISV
Mdn-1       EAIDKLVDVLLEKETLTGDEFRAILSEYTDQPLNTDGDVRIRINDLISV
Xan-1       EAIDKLVDVLLEKETLTGDEFRAILSEYTDQPLNTDGDVRIRINDLISV
Mt-0        EAIDKLVDVLLEKETLTGDEFRAILSEYTDQPLNTDGDVRIRINDLISV
Ct          EAIDKLVDVLLEKETLTGDEFRAILSEYTDQPLNTDGDVRIRINDLISV
C24         EAIDKLVDVLLEKETLTGDEFRAILSEYTDQPLNTDGDVRIRINDLISV
Kas-1       EAIDKLVDVLLEKETLTGDEFRAILSEYTDQPLNTDGDVRIRINDLISV
Col-0       EAIDKLVDVLLEKETLTGDEFRAILSEYTDQPLNTDGDVRIRINDLISV
            *************************************************
```

Supplementary Figure 6.

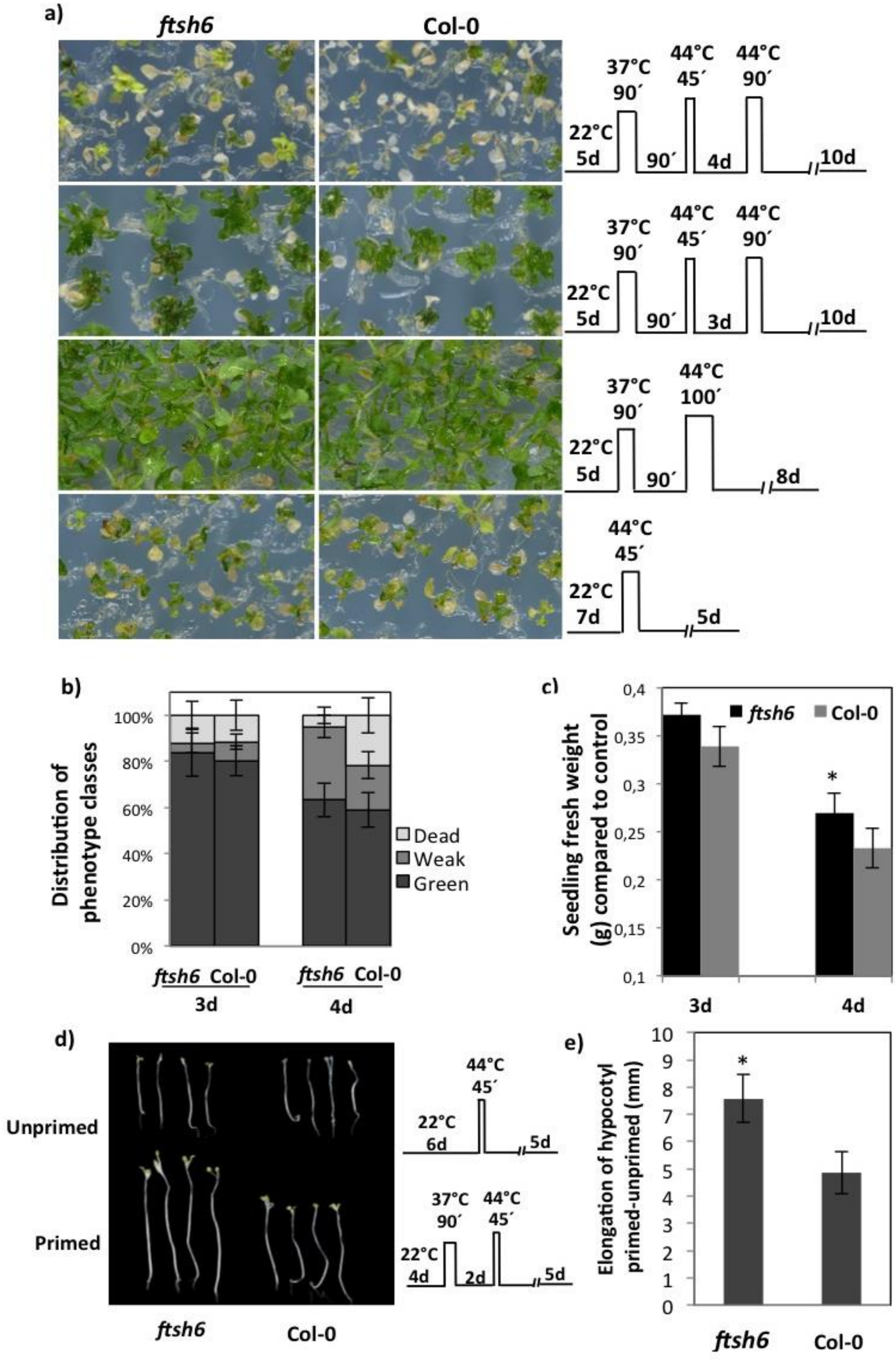

Supplementary Figure 6. Basal and acquired HS tolerance, and thermomemory of *ftsh6* mutants. Seedlings of *ftsh6* mutant and wild-type (Col-0) plants were exposed to different HS regimes (schematically shown on the right of each section). **(a)** Upper middle and top panels: HS triggering stimulus after extended recovery, i.e., 4 and 3 days, respectively, following the priming treatment. Lower middle panel: acquired HS tolerance. Bottom panel: basal HS tolerance; HS applied to 7-day-old seedlings. Following HS, the seedlings were transferred to normal growth condition and photographed after 10 days (upper middle and top panels), 8 days (lower middle panel), or 5 days (bottom panel). **(b)** and **(c)** Quantification of results shown in **a**. **(b)** The percentage of seedlings in different phenotype classes. At 3 d recovery, data for *ftsh6* mutants are not significantly different from Col-0. At 4 d recovery all data for *ftsh6* mutants are different from Col-0 ($p < 0.05$; Student´s *t*-test). **(c)** Seedling fresh weight compared to control plants. Means ± SD are given (n = 5 plates with ~25 seedlings each). **(d)** Hypocotyl elongation assay for *ftsh6* mutants and Col-0 under HS. Six-day-old seedlings were subjected to the HS triggering stimulus without (unprimed) or with (primed) pre-treatment with moderate temperature stress as shown schematically. Photos were taken 5 days after triggering. **(e)** Hypocotyl lengths measured 5 days after recovery from the HS triggering stimulus and compared between primed and unprimed seedlings. Means ± SD are given (n = 7 plates with ~20 seedlings each). Asterisks indicate statistically significant difference ($p < 0.05$; Student´s *t*-test) from the Col-0 control.

Supplementary Figure 7.

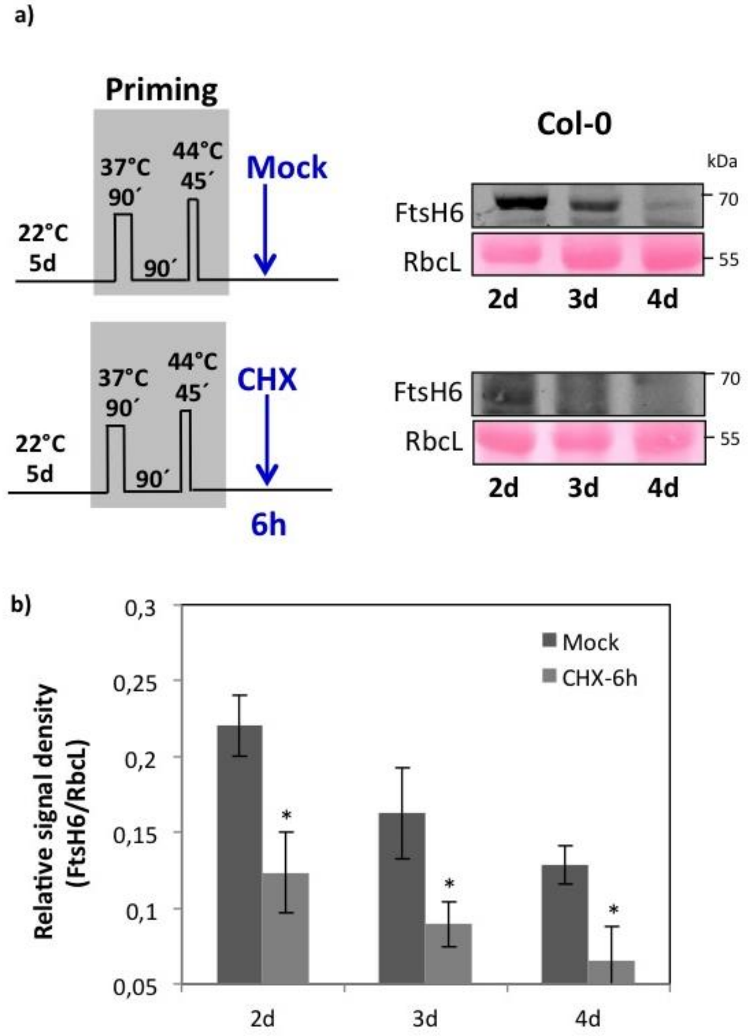

Supplementary Figure 7. The effect of cycloheximide on the accumulation of FtsH6 protein in *Arabidopsis* accession Col-0. **(a)** Immunoblot analyses of FtsH6 protein in Col-0 at days 2, 3 and 4 of the memory phase upon cycloheximide (CHX) and mock (0.1% DMSO) treatment. CHX and mock treatment was applied to seedlings at 6 h into the memory phase, as shown schematically. The seedlings were harvested for immunoblotting at days 2, 3 or 4. kDa, kilo Dalton. **(b)** Signals of the immunoblot analysis were quantified using ImageJ and normalized to the amount of RbcL in the same samples. Mean ± SD are given (n = 3; independent biological replicates each representing a pool of ~120 seedlings grown on 6 plates; gel blots used for the quantification are shown in Supplementary Fig. 10). Asterisks indicate significant difference in the level of FtsH6 between CHX- and mock-treated samples at each indicated time point ($p < 0.05$; Student´s *t*-test).

Supplementary Figure 8.

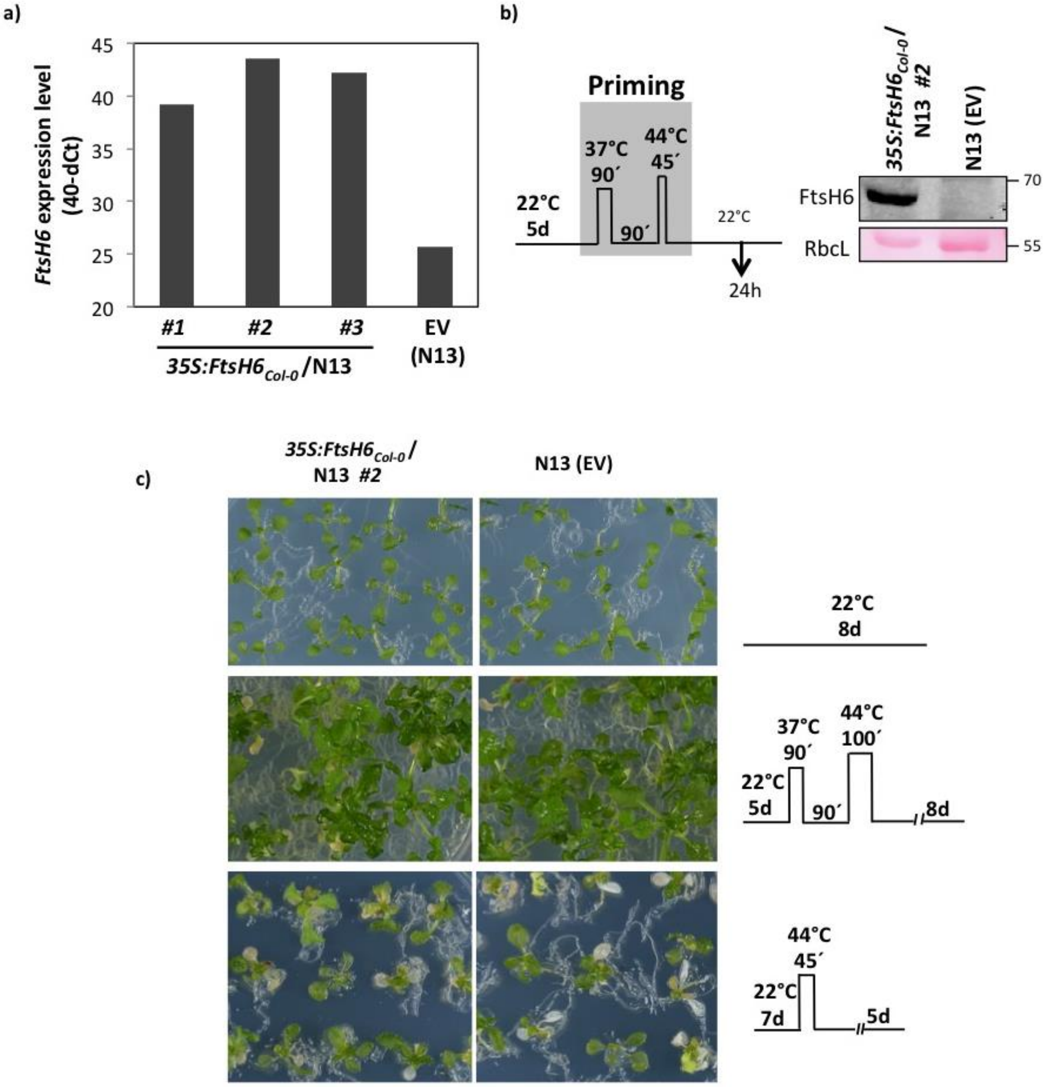

Supplementary Figure 8. ***FtsH6* expression in *35S:FtsH6*$_{Col-0}$/*N13* plants. (a)** Elevated expression of *FtsH6* in *35S:FtsH6*$_{Col-0}$/*N13* plants (lines #1, #2 and #3) compared to N13 empty-vector (EV) control plants. Expression was analysed by qRT-PCR (n = 2). Values are expressed as the difference between an arbitrary value of 40 and dCt, so that high 40-dCt value indicates high gene expression level. **(b)** Immunodetection of FtsH6 protein in *35S:FtsH6*$_{Col-0}$/N13 and N13 EV seedlings exposed to the HS regime shown schematically, using anti-FtsH6 antibody. Note the presence of FtsH6 *protein* in *35S:FtsH6*$_{Col-0}$/N13 plants. RbcL, ribulose-1,5-bis-phosphate carboxylase/oxygenase large subunit (loading control; bottom panel). kDa, kilo Dalton. **(c)** The phenotype of *35S:FtsH6*$_{Col-0}$/N13 and N13 empty-vector (EV) seedlings exposed to different HS regimes (schematically shown on the right of each section in panels).

Supplementary Figure 9.

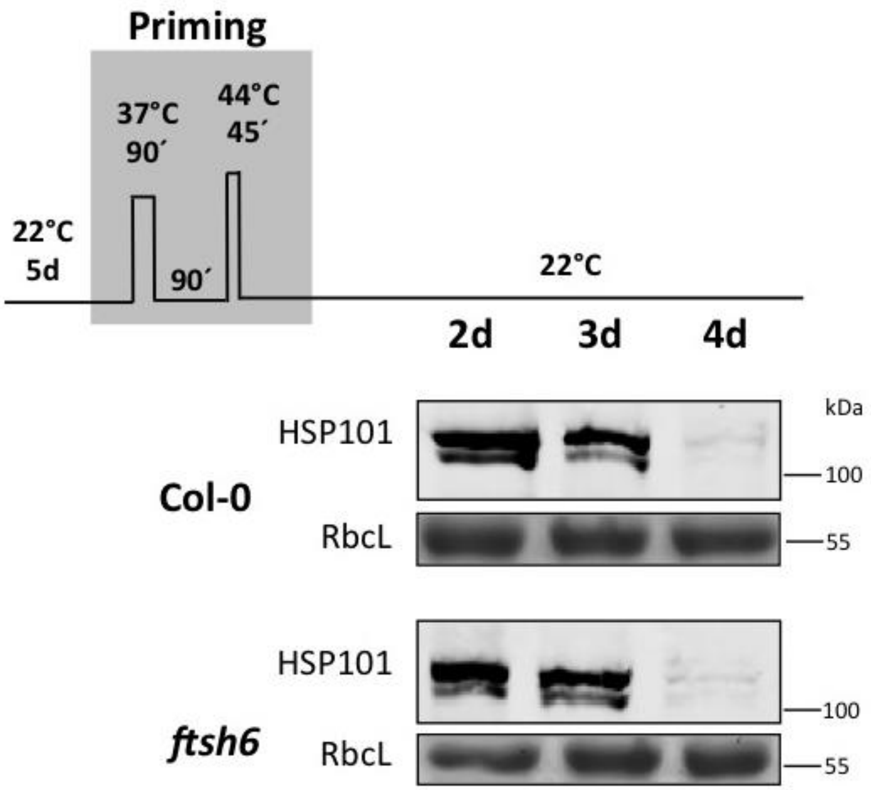

Supplementary Figure 9. Immunodetection of HSP101 protein in Col-0 and *ftsh6.* Immunoblot analysis of HSP101 protein was performed in Col-0 and *ftsh6* seedlings at days 2, 3 and 4 of the memory phase, using anti-HSP101 antibody (Abcam). Note the decline of HSP101 protein level at day 4 of the memory phase with no difference between Col-0 and *ftsh6*. RbcL, ribulose-1,5-bis-phosphate carboxylase/ oxygenase large subunit (loading control). kDa, kilo Dalton.

Supplementary Figure 10.

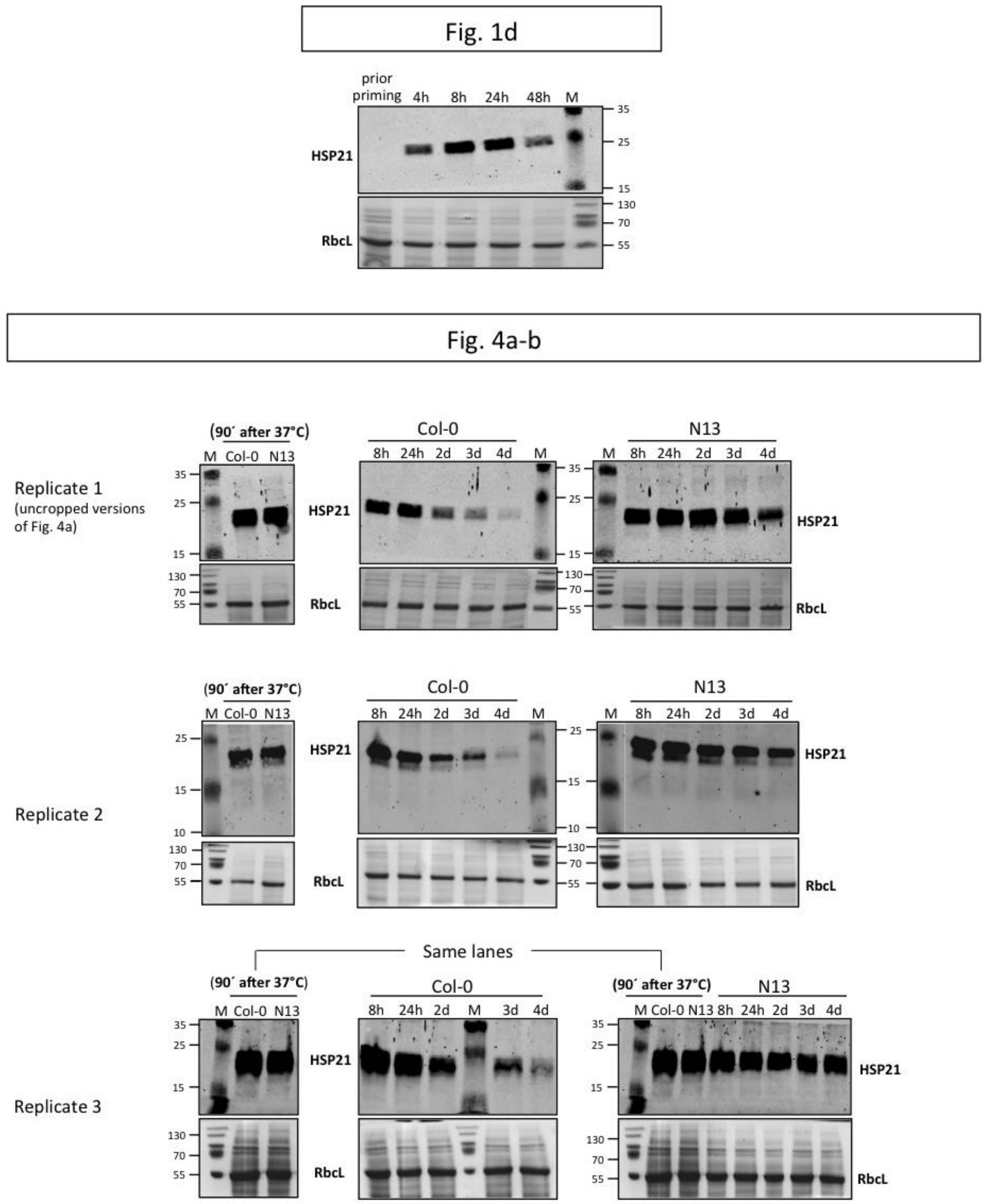

Supplementary Figure 10. Uncropped gel and western blot images presented in this manuscript. Labelling above each image indicates the corresponding figure in the main manuscript or the supplement. M, molecular weight marker. Numbers left and right of images indicate protein molecular weights in kilo Dalton.

Supplementary Figure 10 (continued).

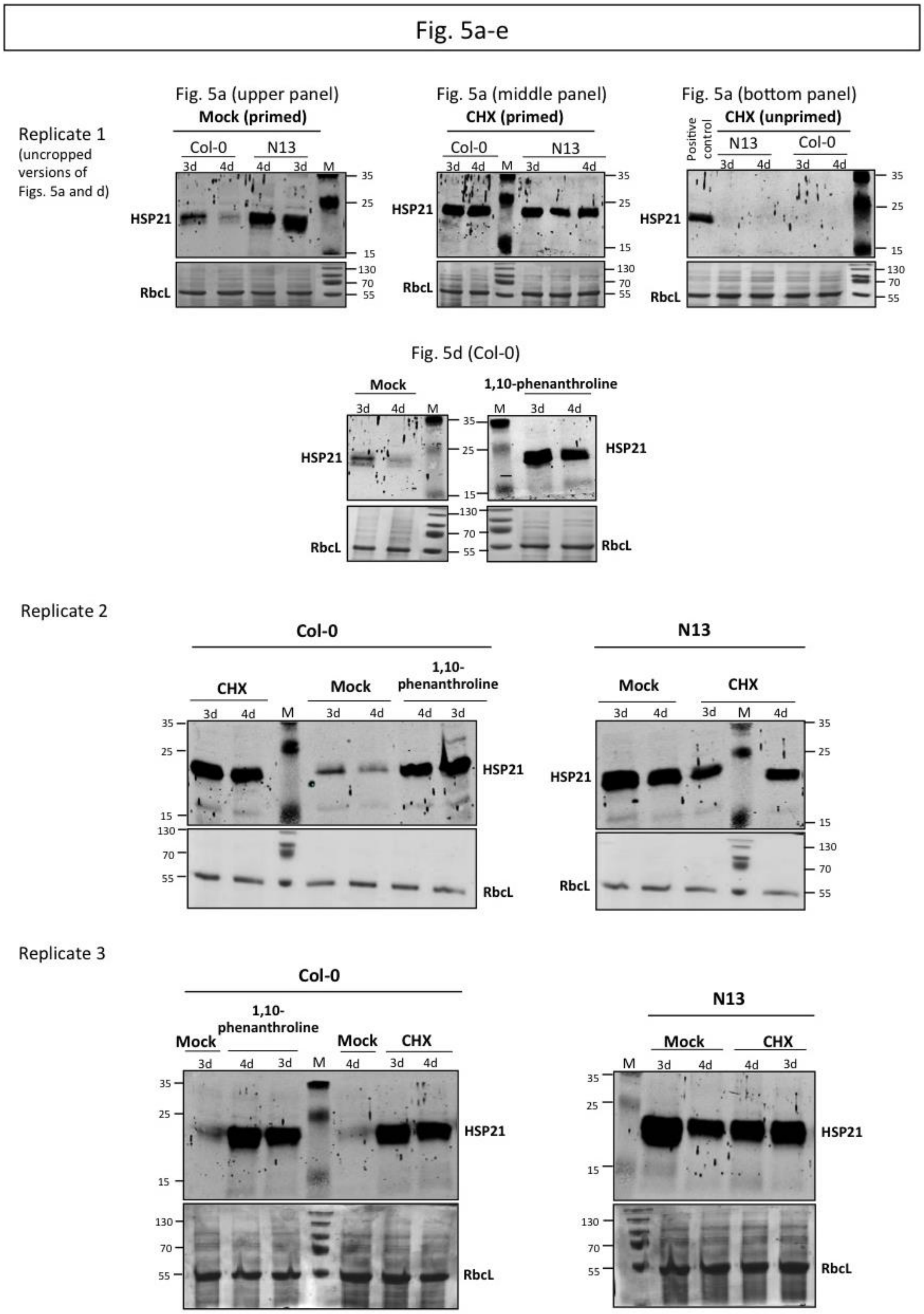

Supplementary Figure 10. Uncropped gel and western blot images presented in this manuscript. Labelling above each image indicates the corresponding figure in the main manuscript or the supplement. M, molecular weight marker. Numbers left and right of images indicate protein molecular weights in kilo Dalton.

Supplementary Figure 10 (continued).

Fig. 6b

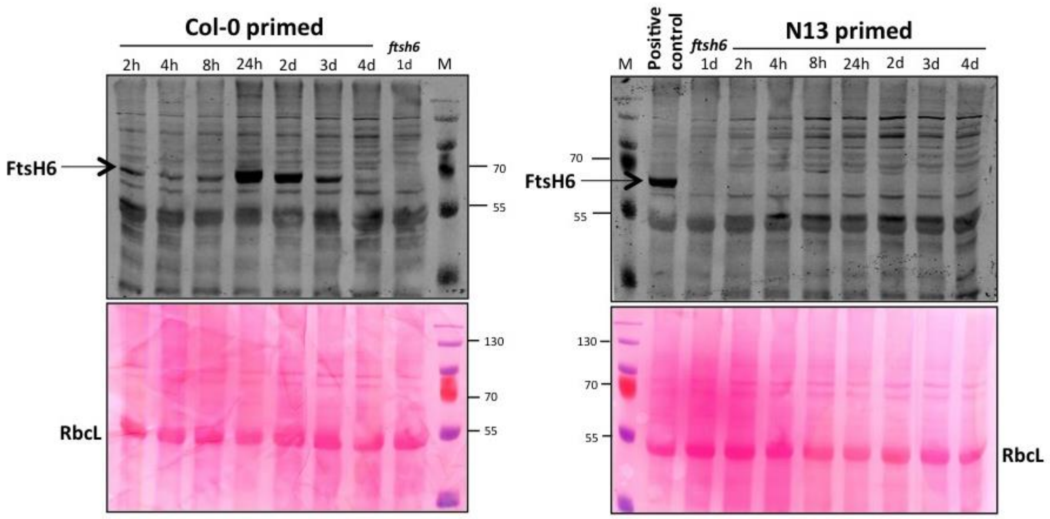

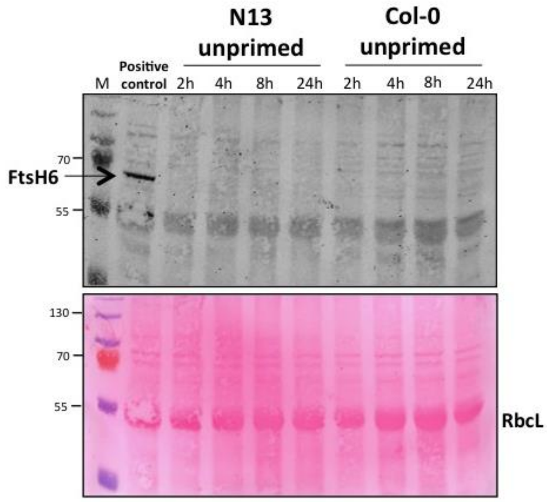

Supplementary Figure 10. Uncropped gel and western blot images presented in this manuscript. Labelling above each image indicates the corresponding figure in the main manuscript or the supplement. M, molecular weight marker. Numbers left and right of images indicate protein molecular weights in kilo Dalton.

Supplementary Figure 10 (continued).

Fig. 6c-d

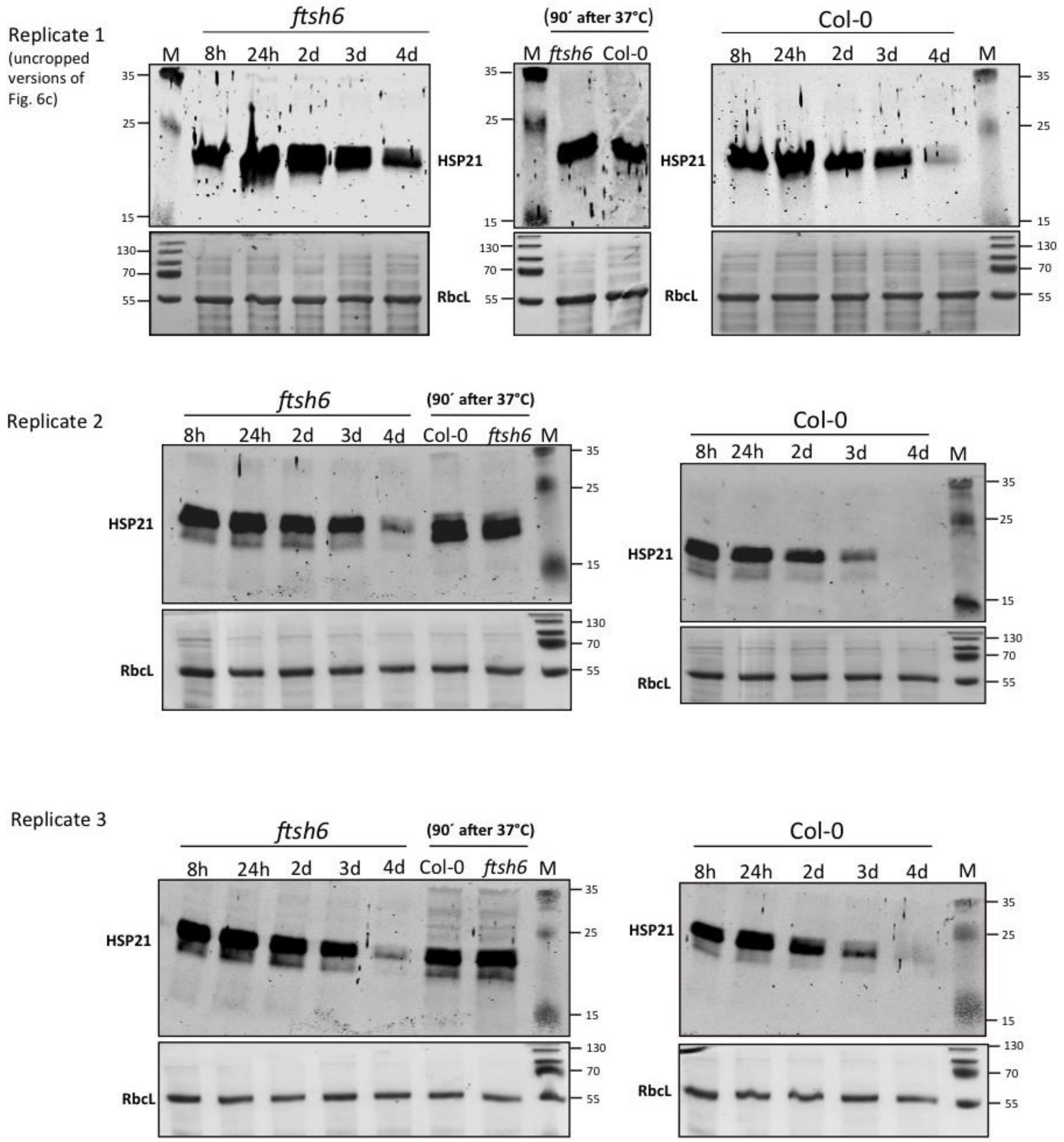

Supplementary Figure 10. Uncropped gel and western blot images presented in this manuscript. Labelling above each image indicates the corresponding figure in the main manuscript or the supplement. M, molecular weight marker. Numbers left and right of images indicate protein molecular weights in kilo Dalton.

Supplementary Figure 10 (continued).

Fig. 7f

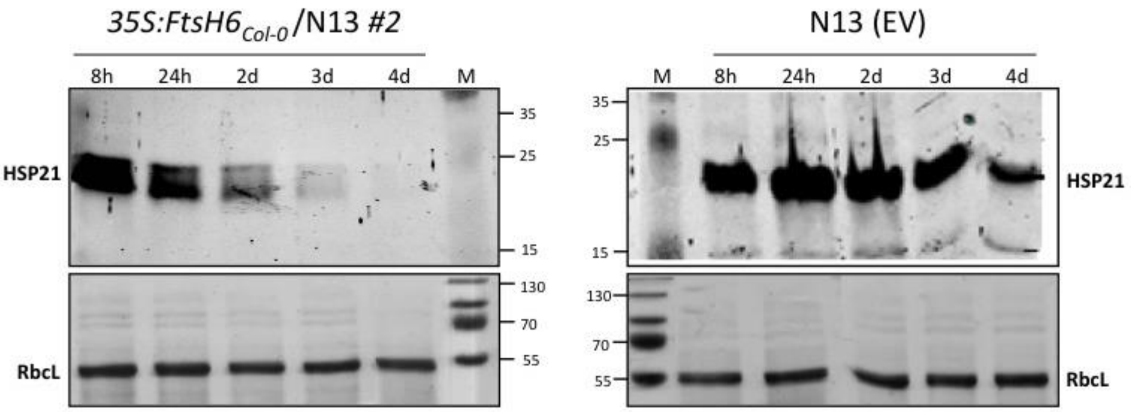

Supplementary Figure 10. Uncropped gel and western blot images presented in this manuscript. Labelling above each image indicates the corresponding figure in the main manuscript or the supplement. M, molecular weight marker. Numbers left and right of images indicate protein molecular weights in kilo Dalton.

Supplementary Figure 10 (continued).

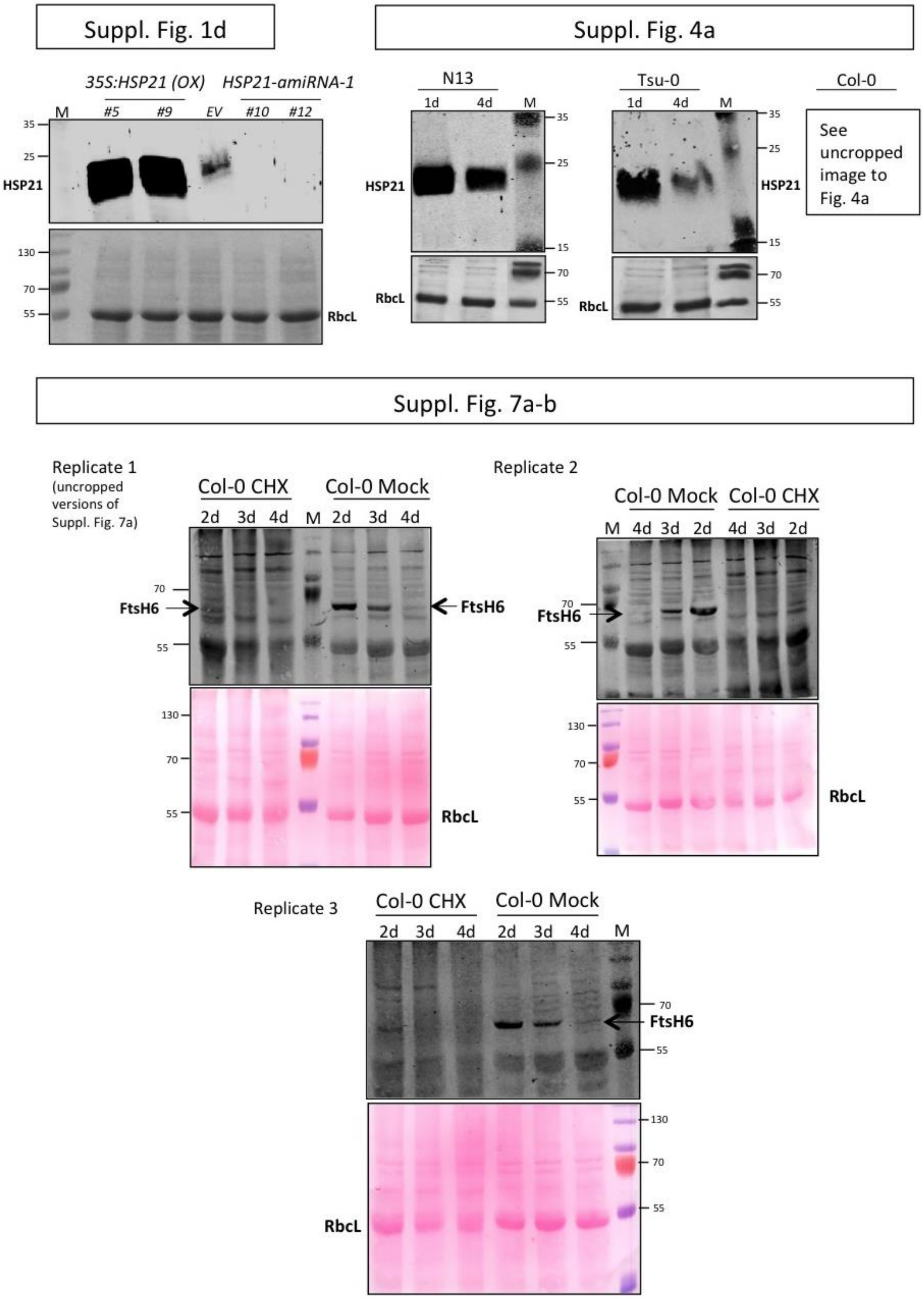

Supplementary Figure 10. Uncropped gel and western blot images presented in this manuscript. Labelling above each image indicates the corresponding figure in the main manuscript or the supplement. M, molecular weight marker. Numbers left and right of images indicate protein molecular weights in kilo Dalton.

Supplementary Figure 10 (continued).

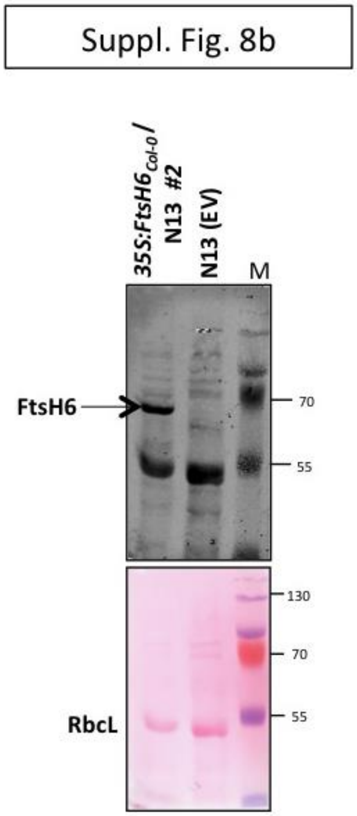

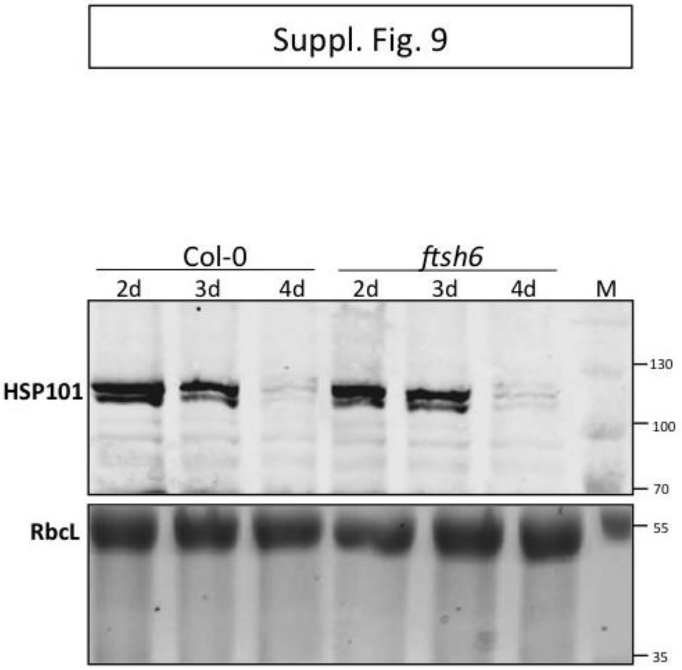

Supplementary Figure 10. Uncropped gel and western blot images presented in this manuscript. Labelling above each image indicates the corresponding figure in the main manuscript or the supplement. M, molecular weight marker. Numbers left and right of images indicate protein molecular weights in kilo Dalton.

Supplementary Table 1. Primer sequences.

**Primers for cloning**	**Sequence**	**Sites added**
35S:HSP21		
HSP21-Forward	GTTTAAACATGGCTTCTACACTCTCATTTGC	added *Pme*I cloning site underlined
HSP21-Reverse	TTAATTAACTACTGAATCTGGACATCGATGA	added *Pac*I cloning site underlined
HSP21-amiRNA		
**amiRNA-1**		
I miR-s-1	GATTAGTATCTAACATTTGTCGCCTCTCTTTTGTATTCCA	
II miR-a-1	AGGCGACAAATGTTAGATACTAATCAAAGAGAATCAATGA	
III miR*s-1	AGGCAACAAATGTTACATACTATTCACAGGTCGTGATATG	
IV miR*a-1	GAATAGTATGTAACATTTGTTGCCTACATATATATTCCTA	
**amiRNA-II**		
I miR-s-2	GATGTATCTAACATTTGTCGCATCTCTCTTTTGTATTCCA	
II miR-a-2	AGATGCGACAAATGTTAGATACATCAAAGAGAATCAATGA	
III miR*s-2	AGATACGACAAATGTAAGATACTTCACAGGTCGTGATATG	
IV miR*a-2	GAAGTATCTTACATTTGTCGTATCTACATATATATTCCTA	
**amiRNA-III**		
I miR-s-3	GATATAATGTTGATCGAGTCCTATCTCTCTTTTGTATTCC	
II miR-a-3	GATAGGACTCGATCAACATTATATCAAAGAGAATCAATGA	
III miR*s-3	GATAAGACTCGATCATCATTATTTCACAGGTCGTGATATG	
IV miR*a-3	GAAATAATGATGATCGAGTCTTATCTACATATATATTCCT	
A Primer (universal for making amiRNA)	GTTTAAACCTGCAAGGCGATTAAGTTGGGTAAC	added *Pme*I cloning site underlined
B primer (universal for making amiRNA)	TTAATTAAGCGGATAACAATTTCACACAGGAAACAG	added *Pac*I cloning site underlined

qRT-PCR primers

**Gene**	**Forward**	**Reverse**
Actin2	TCCCTCAGCACATTCCAGCAGAT	AACGATTCCTGGACCTGCCTCATC
HSP21	TGGACGTCTCTCCTTTCGGATTG	TTTGTCGCATCGTCCTCATTGG
FtsH6	GCCGGAATGGAAGGGACAAAGATG	ATCATGACCCTCCGTCAAAGTCG
